# Pan-Cancer Methylated Dysregulation of Long Non-coding RNAs Reveals Epigenetic Biomarkers

**DOI:** 10.3389/fcell.2022.882698

**Published:** 2022-05-27

**Authors:** Ning Zhao, Maozu Guo, Chunlong Zhang, Chunyu Wang, Kuanquan Wang

**Affiliations:** ^1^ School of Life Science and Technology, Harbin Institute of Technology, Harbin, China; ^2^ School of Electrical and Information Engineering, Beijing University of Civil Engineering and Architecture, Beijing, China; ^3^ College of Information and Computer Engineering, Northeast Forest University, Harbin, China; ^4^ School of Computer Science and Technology, Harbin Institute of Technology, Harbin, China

**Keywords:** lncRNA, pan-cancer, DNA methylation, biomarker, ceRNA

## Abstract

Different cancer types not only have common characteristics but also have their own characteristics respectively. The mechanism of these specific and common characteristics is still unclear. Pan-cancer analysis can help understand the similarities and differences among cancer types by systematically describing different patterns in cancers and identifying cancer-specific and cancer-common molecular biomarkers. While long non-coding RNAs (lncRNAs) are key cancer modulators, there is still a lack of pan-cancer analysis for lncRNA methylation dysregulation. In this study, we integrated lncRNA methylation, lncRNA expression and mRNA expression data to illuminate specific and common lncRNA methylation patterns in 23 cancer types. Then, we screened aberrantly methylated lncRNAs that negatively regulated lncRNA expression and mapped them to the ceRNA relationship for further validation. 29 lncRNAs were identified as diagnostic biomarkers for their corresponding cancer types, with lncRNA *AC027601* was identified as a new KIRC-associated biomarker, and lncRNA *ACTA2-AS1* was regarded as a carcinogenic factor of KIRP. Two lncRNAs *HOXA-AS2* and *AC007228* were identified as pan-cancer biomarkers. In general, the cancer-specific and cancer-common lncRNA biomarkers identified in this study may aid in cancer diagnosis and treatment.

## Introduction

Cancer is a general term that refers to malignant tumors, and its world-wide incidence and mortality have been high for many years. In 2020, there were 19.29 million new cancer cases and 9.96 million cancer deaths world-wide (Sung et al., 2021). There is no cure for cancer at the moment. Not only do different types of cancer have common biological characteristics such as abnormal cell differentiation and proliferation, lack of growth control, invasion and metastasis, but they also have many specifically biological characteristics, respectively. Therefore, it is necessary to conduct pan-cancer research on a variety of cancer types to ascertain the similarities and differences in molecular characteristics across different cancers.

DNA methylation is an important epigenetic modification that plays an important role in many physiological processes (Martin-Subero, 2011; Li et al., 2013; Neri et al., 2017), and its aberrant behavior can result in gene instability, proto-oncogene activation and tumor suppressor gene inactivation (Li et al., 2012; Hou et al., 2021). Aberrant DNA methylation occurs in almost all cancers, where unmethylated promoters become methylated or methylated sequences lose their methylation. Focusing on the pan-cancer analysis of DNA methylation can inform research and therapy. Saghafinia et al. (Saghafinia et al., 2018) described an algorithmic strategy for identifying pan-cancer-related DNA methylation alteration affecting gene expression. Numerous DNA methylation dysregulation were discovered to be associated with patient prognosis and therapeutic response. Methylated research in cancer patients has been shown to improve and maintain the efficiency of cancer treatment (Pauken et al., 2016; Sen et al., 2016; Marwitz et al., 2017).

Long non-coding RNAs (lncRNA) are non-coding RNAs with a length of more than 200 nucleotides. In comparison to protein-coding genes, they have a high degree of tissue specificity in their expression (Cabili et al., 2011). LncRNAs play an important role in the occurrence and development of cancer and many other complex diseases (Zhou et al., 2019). LncRNAs are associated with practically every major cancer type and contribute to all ten hallmarks of cancer (Huarte, 2015; Bartonicek et al., 2016; Esposito et al., 2019; Bao et al., 2020). Additionally, lncRNAs play an important role in immune regulation (Chen et al., 2017; Zhang et al., 2021b). Lin et al. (Lin and Yang, 2018) explored the mechanisms by which lncRNAs regulated cellular responses to extracellular signals and their clinical potential as diagnostic indicators, stratification markers, and therapeutic targets for combinatorial treatments. Zhang et al. (Zhang et al., 2018) identified lncRNA *MT1JP* as a ceRNA for the tumor suppressor *FBXW7* in gastric cancer by demonstrating competitive binding with MiR-92A-3p. Xu et al. (Xu et al., 2019) discovered that lncRNA *SNHG6* regulated the expression of the oncogene *EZH2* in colorectal cancer *via* the ceRNA sponge-associated with MiR-26a/b and MiR-214. Chen et al. (Chen et al., 2019) discovered that the lncRNA *PVT1* promoted tumor development in gallbladder cancer by regulating the miR-143/HK2 axis. Wang et al. (Wang et al., 2017) identified the lncRNA *HOXD-AS1* as a ceRNA that regulated *SOX4* and promoted liver cancer metastasis. The above studies established the role of lncRNAs in corresponding cancer types, and identified the cancer-associated lncRNAs for each cancer type (Dong et al., 2019). Pan-cancer analysis of lncRNAs can help in identifying the similarities and differences between distinct cancer types and identifying potential therapeutic targets for cancer treatment.

Numerous pan-cancer studies have been carried out on lncRNAs. Li et al. (Yongsheng Li et al., 2020) identified multiple pan-cancer immune-associated lncRNAs as potential oncogenic biomarkers. Martens-Uzunova et al. (Martens-Uzunova et al., 2014) summarized the role of lncRNAs in the diagnosis and treatment of urinary tumors, and concluded that lncRNAs could be used as new biomarkers for prostate cancer, kidney cancer and bladder cancer. Zhang et al. (Zhang et al., 2021) identified clinically distinct tumor subtypes by characterizing pan-cancer lncRNA modifiers of the immune microenvironment. Bao et al. (Bao et al., 2021) proposed a framework for identifying lncRNA signatures associated with pan-cancer prognosis.

Previous studies have established a correlation between lncRNAs and epigenetic regulation (Spizzo et al., 2012; Xu et al., 2018), suggesting that they regulates chromatin state and epigenetic inheritance (Tsai et al., 2010). Lu et al. (Lu et al., 2020) found that DNA methylation-mediated lncRNA activation improved temozolomide resistance in glioblastoma, implying that *SNHG12* could be a therapeutic target for overcoming temozolomide tolerance.

Detecting the dynamic pattern of lncRNA methylation during cancer development across pan-cancer may help highlight epigenetic changes and aid in cancer diagnosis and treatment. Yang et al. (Yang et al., 2021) presented a novel integrative analysis framework, termed MeLncTRN for integrating data on gene expression, copy number variation, methylation and lncRNA expression. They identified epigenetically-driven lncRNA-gene regulation circuits across 18 cancer types. Wei et al. (Wei et al., 2019) constructed a systematic biological framework to evaluate the co-methylation events between two lincRNAs in nine cancer types. The lincRNA prognostic signatures were identified to significantly correlate with overall survival in cancers. Wang et al. (Wang et al., 2018) characterized the epigenetic landscape of genes encoding lncRNAs associated with pan-cancer and identified *EPIC1* as an oncogenic lncRNA. Xu et al. (Xu et al., 2021) constructed networks of lncRNA-associated dysregulated ceRNA across eight cancer types. They screened nine pan-cancer epigenetically related lncRNAs.

However, no research has been conducted to systematically compare methylation changes of lncRNAs in pan-cancer to identify the specific methylation-related lncRNAs. In this study, we used pan-cancer lncRNA methylation data from the TCGA to examine the lncRNA methylation patterns of 23 cancer types and identified differentially methylated lncRNAs (DMlncs). Subsequently, we examined differentially methylated lncRNAs from different cancer types to identify cancer-specific and cancer-common differentially methylated lncRNAs. Further, combining lncRNA expression data with survival data, lncRNAs with a negative correlation between methylation and expression dysregulation were found as diagnostic biomarkers for each cancer. Finally, the lncRNAs were mapped into the ceRNA network to establish ceRNA relationships with mRNAs confirming their important roles in cancer.

## Materials and Methods

### Data

Pan-cancer DNA methylation data for lncRNAs from Infinium 450k arrays were downloaded from the TCGA database. The cancer types with normal samples were selected, and a total of 7,634 tumor samples and 746 normal samples from 23 cancer types were retained. Table 1 shows the number of tumor and normal samples, as well as the number of lncRNAs. The methylation status of each probe in each sample was measured using the β-value (Aryee et al., 2014). The β-value denoted the ratio of methylation intensity of the probe to total intensity, with a range of 0 (low methylation) to 1 (high methylation). The probes with β-values greater than 0 in more than 50% of the samples were retained in the methylation profile for each cancer, and the missing values were filled with the average of all non-zero values on the probes. The average β-value of the promoter region was used to determine the methylation level of each lncRNA.

**TABLE 1:** The number of samples and lncRNAs for each cancer in methylation data.

Cancer	No. of tumor samples	No. of normal samples	No. of lncRNAs
BLCA	412	21	4,317
BRCA	783	96	4,314
CESC	307	3	4,314
CHOL	36	9	4,309
COAD	296	38	4,317
ESCA	185	16	4,317
GBM	141	2	4,317
HNSC	528	50	4,317
KIRC	319	160	4,317
KIRP	275	45	4,317
LIHC	377	50	4,317
LUAD	458	32	4,316
LUSC	370	42	4,317
PAAD	184	10	4,317
PCPG	179	3	4,315
PRAD	498	50	4,317
READ	98	7	4,316
SARC	261	4	4,310
SKCM	470	2	4,317
STAD	395	2	4,314
THCA	507	56	4,314
THYM	124	2	4,316
UCEC	431	46	4,314

Expression data of lncRNAs and mRNAs for 13 cancer types were downloaded from the TANRIC and the TCGA databases, respectively (Li et al., 2015). The number of tumor and normal samples and the number of lncRNAs and mRNAs are shown in Supplementary Tables S1, S2. Each lncRNA and mRNA expression value was defined as its reads per kilobase per million mapped reads (RPKM) (Mortazavi et al., 2008). Subsequently, we transformed the expression data by log2 (RPKM+1), reserved the lncRNAs and mRNAs with expression values in more than 70% of the samples, and filled their missing values using the average expression values of these RNAs in the samples.

The expression data of mRNA and lncRNA was downloaded from different databases, so we got the human gene annotation files from the GENCODE database (https://www.gencodegenes.org/) to obtain the corresponding relations of ENSG IDs and gene symbols. Then, using Entrez IDs as the main reference, different versions of human gene names (Entrez IDs, gene symbols and ENSG IDs) were converted to standard human gene names.

### Identification of DMlncs

The DMlncs for each cancer were first screened using the following formula  Δβ :

$$\Delta \beta =\left|{\bar{\beta }}_{t}-{\bar{\beta }}_{n}\right|$$ (1)

Where, ${\bar{\beta }}_{t}$ and ${\bar{\beta }}_{n}$ denoted the average level of methylation in tumor and normal samples, respectively. Δβ was the subtraction difference in average methylation levels between tumor and normal samples. lncRNAs with Δβ >0.1 were selected as candidates for DMlncs.

Additionally, the “limma” package (Ritchie et al., 2015) in R language was used to measure the degree of difference between tumor and normal samples. The lncRNAs with | $log_{2}\left(fold\;change\right)$ |>1 (PCAWG Transcriptome Core Group et al., 2020) and FDR<0.05 were identified as DMlncs.

### Identification of Differentially Expressed lncRNAs

The “limma” package was used to calculate differential expression between tumor and normal samples for lncRNA expression data. We took the lncRNAs with | $log_{2}\left(fold\;change\right)$ |>1 and FDR<0.05 as differentially expressed lncRNAs.

### Functional Enrichment Analysis

The GREAT software (McLean et al., 2010) was used to conduct functional enrichment analysis on lncRNAs. We took the lncRNA BED data as input. The lncRNA BED information included the chromosome, start site and end site extracted from GENCODE database. Gene ontology (GO) functions of the output results were selected for subsequent analysis.

### Recognition of ceRNAs

LncRNA-miRNA and mRNA-miRNA targeted relationships were downloaded from the ENCORI platform (Li et al., 2014). For each pair of lncRNA and mRNA, the intersection of their target miRNAs should be more than two (Zhang et al., 2021a). The intersections of their miRNA lists were subjected to the hypergeometric test, and the lncRNA-mRNA pairs with a *p*-value less than 0.05 were considered. A total of 4,739,668 pairs were obtained.

Subsequently, the Pearson correlation coefficient between lncRNA expression and mRNA expression was calculated. LncRNA-mRNA pairs with Pearson correlation coefficient >0.3 and *p*-value<0.05 were selected as ceRNAs. Supplementary Table S3 shows the number of lncRNA-mRNA pairs, lncRNAs and mRNAs in each cancer.

### Survival Analysis

Survival analysis of patients was carried out using the “survival” package in R language, in which the maxstat model was used to evaluate the best cut-off point to divide high-risk and low-risk groups. Kaplan-Meier curves were then drawn to depict the survival of patients of high-risk and low-risk groups.

### Immunological Score

Three scores were used to assess the immunological effect of lncRNAs: Major Histocompatibility Complex (MHC), Cytolytic Activity (CYT) and Cytotoxic T Lymphocyte (CTL). The MHC score of each sample was calculated as:

$$Score_{MHC_{i}}=\left(\sum exp_{n}\right)/9$$ (2)

Where *i* denoted the sample, $exp_{n}$ represented the expression of gene *n*, and *n* was one of the nine genes (*HLA-A, PSMB9, HLA-B, PSMB8, HLA-C, B2M, TAP2, NLRC5,* and *TAP1*). These nine genes had a strong correlation and were the core gene set of MHC-I (Rooney et al., 2015; Lauss et al., 2017).

The CYT score of each sample was calculated as follows:

$$Score_{CYT_{i}}=\left(exp_{GZMA}+exp_{PRF1}\right)/2$$ (3)

In which, *i* represented the sample, $exp_{GZMA}$ and $exp_{PRF1}$ represented the expression of *GZMA* and *PRF1*, respectively. These two genes were key factors of cytolysis and were upregulated in activated CD8+T cells and strongly responded to *CTLA4* and *PDCD1* immunotherapy (Narayanan et al., 2018).

The CTL score of each sample was calculated as follows:

$$Score_{CTL_{i}}=\left(exp_{GZMA}+exp_{PRF1}+exp_{GZMB}\right)/3.$$ (4)

In which, *i* represented the sample, $exp_{GZMA}$ , $exp_{PRF1}$ and $exp_{GZMB}$ represented the expression of *GZMA*, *PRF1*, and *GZMB*, respectively. These three genes were important factors to measure T cell toxicity and immune cell effector function (Basu et al., 2016).

## Results

### The Workflow of DMlncs Identification

**FIGURE 1:**
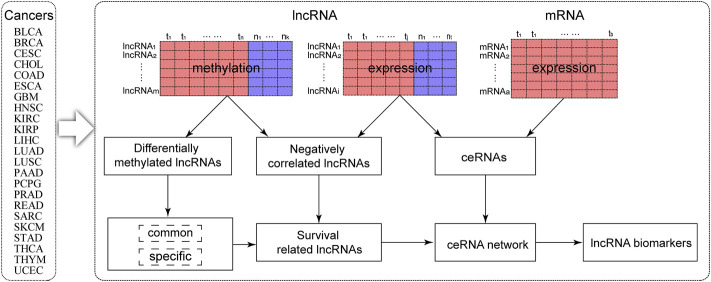
The framework for identifying the lncRNA methylation biomarkers.

In this study, the identification flow of methylation-related lncRNA biomarkers for pan-cancer is shown in Figure 1. We conducted a study on a total of 23 cancer types. Firstly, DMlncs between tumor and normal samples were obtained using lncRNA methylation data. Specific and common lncRNAs were identified for pan-cancer. Subsequently, lncRNA methylation data and lncRNA expression data were integrated to identify DMlncs whose methylation changes were negatively correlated with expression changes. Following that, prognostic lncRNAs were screened and mapped into the ceRNA network. The ceRNA network was constructed by combining lncRNA and mRNA expression data. Finally, pan-cancer lncRNA biomarkers were identified by analyzing the lncRNAs of the ceRNA network.

### Identification of Pan-Cancer DMlncs

**FIGURE 2:**
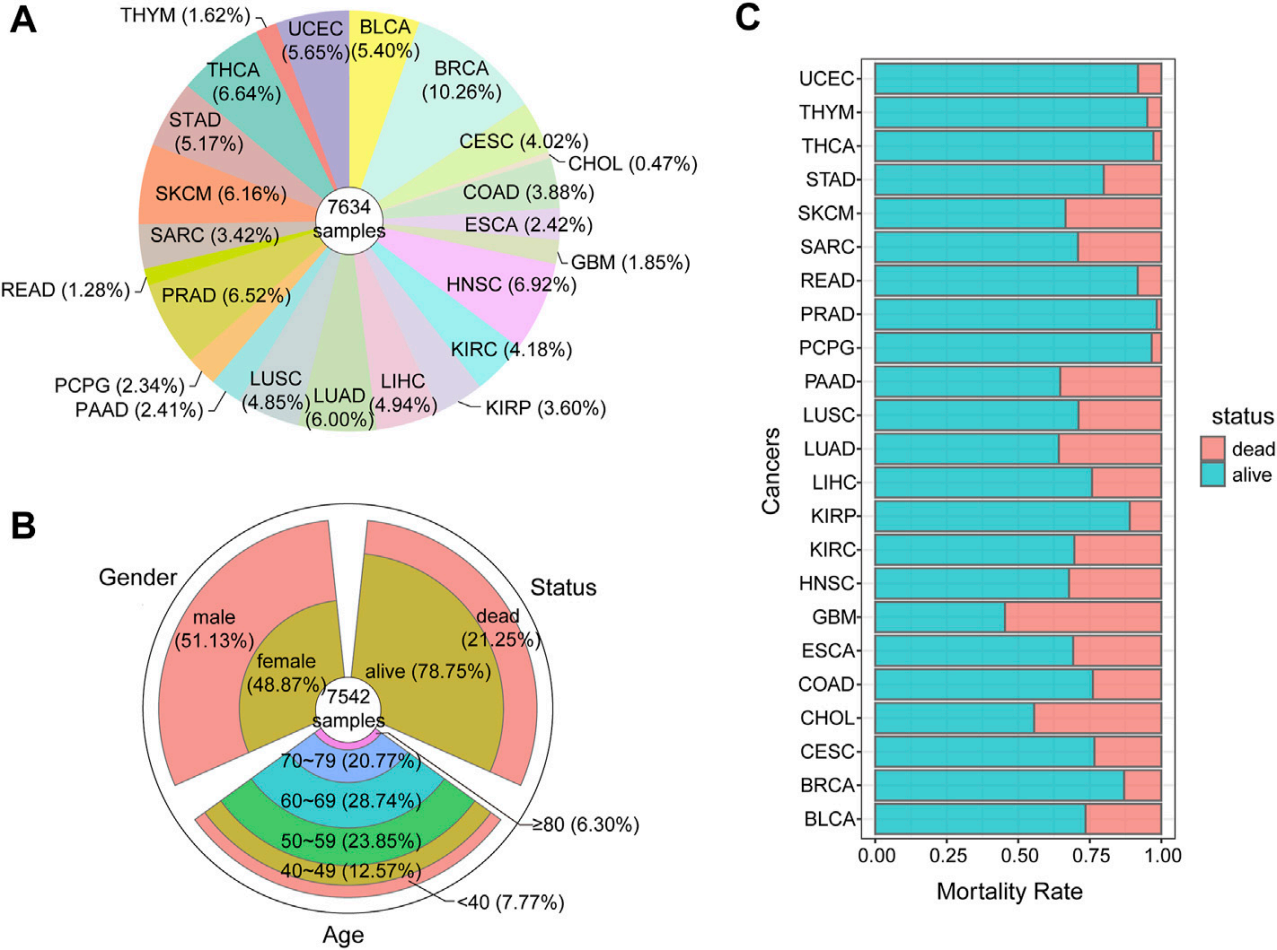
Characteristics of pan-cancer tumor samples **(A)**The proportion of samples collected for each cancer **(B)**The clinical information of tumor samples **(C)**The mortality rate of each cancer.

To identify DMlncs, we downloaded cancer methylation profiles of 23 cancer types from the TCGA, including both tumor and normal samples. The data of 7,634 tumor samples and 746 normal samples was downloaded. The proportion of tumor samples for each cancer is shown in Figure 2A. BRCA had the largest number of samples, which was nearly double that of other cancer types, while CHOL had the lowest number of samples. A total of 7,542 tumor samples had corresponding clinical data of the patients. As shown in Figure 2B, the age, sex, and survival status of patients were analyzed. The male to female ratio of the patients was 1:1, and the age was concentrated among the elderly, which was consistent with the law of the general onset age of cancer. The majority of patients survived following treatment. Figure 2C shows the mortality rate of tumor patients, three-quarters of the patients survived after surgery. GBM had the highest mortality rate, followed by CHOL. PRAD had the lowest mortality rate.

After standardizing the data, DMlncs for each cancer were identified. The number of DMlncs is shown in Table 2. A total of 2,286 DMlncs were obtained. The majority of the cancer types had a large number of DMlncs, and only a few cancer types had a small number of DMlncs.

**TABLE 2:** The number of DMlncs of each cancer.

Cancer	Total lncRNAs	Down-methylated lncRNAs	Up-methylated lncRNAs	Cancer-specific up-methylated	Cancer-specific down-methylated
BLCA	1,126	931	195	2	45
BRCA	753	411	342	23	9
CESC	395	99	296	20	8
CHOL	433	80	353	65	23
COAD	762	464	298	28	14
ESCA	569	263	306	28	1
GBM	48	7	41	13	2
HNSC	854	581	273	9	12
KIRC	435	286	149	5	24
KIRP	406	154	252	57	4
LIHC	1,074	936	138	10	114
LUAD	583	349	234	5	1
LUSC	932	674	258	11	18
PAAD	344	150	194	11	9
PCPG	174	112	62	26	47
PRAD	604	236	368	77	13
READ	575	375	200	5	6
SARC	7	1	6	2	1
SKCM	74	0	74	22	0
STAD	8	7	1	0	7
THCA	118	99	19	3	5
THYM	3	0	3	2	0
UCEC	1,015	651	364	32	47

**FIGURE 3:**
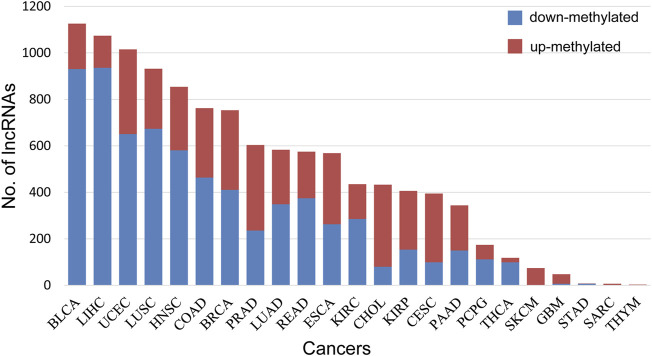
The percentage of up-methylated and down-methylated lncRNAs for each cancer. Blue represents the number of down-methylated lncRNAs, and red represents the number of up-methylated lncRNAs.

Additionally, DMlncs were divided into up-methylated and down-methylated groups. Up-methylated lncRNAs were those whose methylation level was elevated in cancer samples compared with normal samples, while down-methylated lncRNAs were the inverse. Table 2 shows the number of patients in each of the two groups. There were overlaps in the genes of different cancers. In total, there were 1,229 DMlncs in the up-methylated group and 1,654 DMlncs in the down-methylated group. Among them, 597 lncRNAs were up-methylated in some cancers but down-methylated in some other cancers, demonstrating uneven regulation tendencies across different cancers. As shown in Figure 3, the proportion of up-methylated and down-methylated lncRNAs varied between cancer types. There were 13 types of cancer had a higher number of down-methylated lncRNAs and ten types of cancer had a higher number of up-methylated lncRNAs. The first few cancer types of most DMlncs had obvious higher number of down-methylated lncRNAs and most of the other cancer types had more up-methylated lncRNAs. Certain cancer types contained only a single type of DMlncs. For example, all 74 DMlncs in SKCM were up-methylated, and all three DMlncs in THYM were up-methylated as well.

### Cancer-Specific lncRNA Biomarkers

**FIGURE 4:**
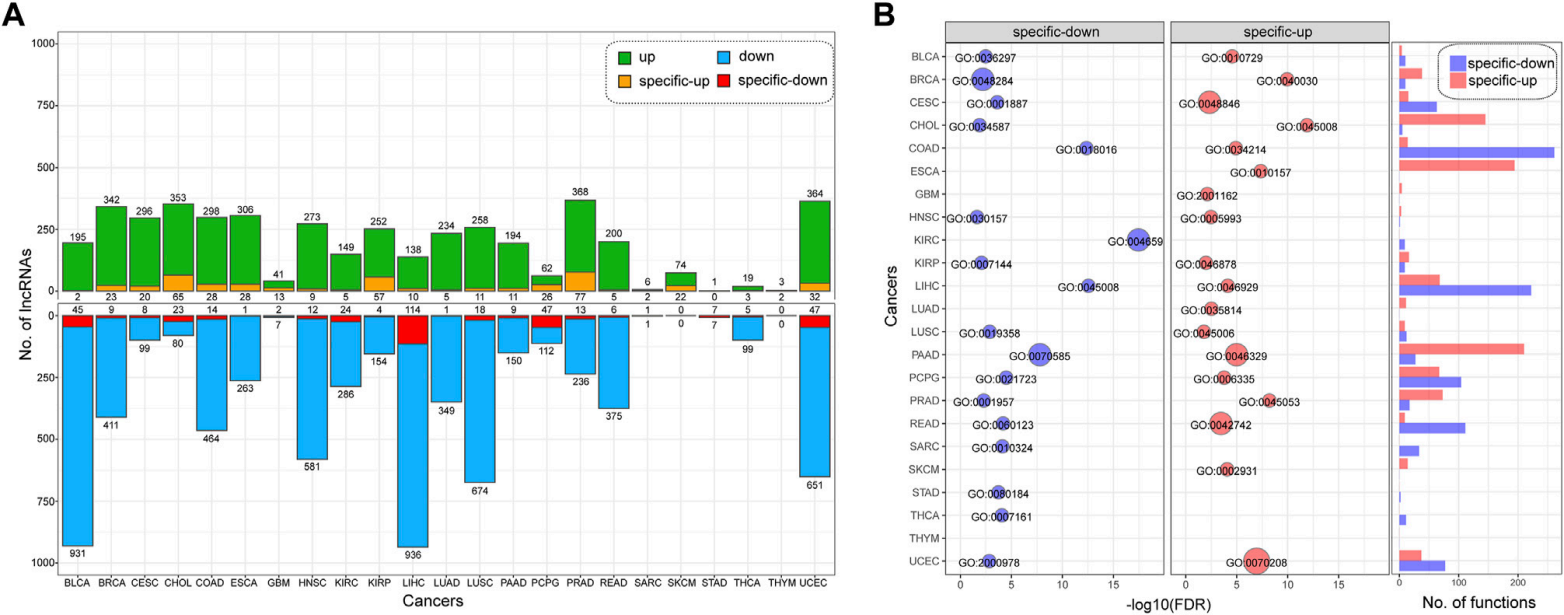
The cancer-specific lncRNAs with differential methylation **(A)**The number and proportion of cancer-specific lncRNAs. Green represents up-methylated lncRNAs, blue represents down-methylated lncRNAs, yellow represents specifically up-methylated lncRNAs, and red represents specifically down-methylated lncRNAs **(B)** The functions of specific lncRNAs in each cancer type. Red represents the functions of up-methylated lncRNAs, while blue represents the functions of down-methylated lncRNAs.

As a complex disease, cancer has a high heterogeneity and distinct pathogenesis. In this study, we searched for specific DMlncs for each cancer. Figure 4A shows the proportion of cancer-specific lncRNAs, and only a fraction of the DMlncs were cancer-specific. LIHC had the most specifically down-methylated lncRNAs, whereas PRAD had the most specifically up-methylated lncRNAs. The proportion of both specifically up and down lncRNAs in PCPG was about 42%. SARC, STAD, and THCA had DMlncs less than ten, preventing them from being compared with other cancers in terms of lncRNA proportion. Except for SARC, STAD, and THCA, PCPG had the highest proportion of specific lncRNAs. STAD had no up-methylated lncRNAs, while all its seven down-methylated lncRNAs were specific. SARC possessed a single down-methylated lncRNA, and it was specific. Two of the three DMlncs of THYM were specific. Supplementary Table S4 shows the specific DMlncs of each cancer.

Subsequently, we performed functional enrichment analysis of specifically up-methylated and down-methylated lncRNAs in each cancer. For each cancer, the functions enriched by specifically up-methylated and down-methylated lncRNAs were analyzed separately, and the number of functions enriched by each cancer is shown on the right side of Figure 4B. There were significant differences in the number of enriched functions for the cancers. LIHC, COAD, and PAAD enriched in more than 200 functions, while the enriched functions of THYM, STAD, and HNSC were less than five. There also a difference between the number of functions of up-methylated and down-methylated lncRNAs for each cancer. The specifically down-methylated lncRNAs of COAD were enriched in the majority of functions (261), whereas up-methylated lncRNAs of COAD were enriched in only a few functions (14), and LIHC demonstrated a similar pattern. The specifically up-methylated lncRNAs of ESCA were found to be enriched in a variety of functions (194), but the down-methylated lncRNAs were enriched in no functions. The details of enriched functions are shown in Supplementary Table S5.

Among the functions, we selected the most significant function for each group and displayed them on the left side of Figure 4B. The function names, enrichment *p*-values and other information are shown in Table 3. The lncRNAs of most cancer types were enriched in the “regulation” or “response” functions. Both the up-methylated group of CHOL and the down-methylated group of LIHC were enriched in “depyrimidine”. The specifically up-methylated lncRNAs of GBM were enriched in “methylation”, the specifically down-methylated lncRNAs of COAD were enriched in “dimethylation”, and the specifically up-methylated lncRNAs of BRCA were enriched in “epigenetic”. These results established the important role of lncRNAs in the epigenetic process.

**TABLE 3:** The functions of specific lncRNAs for each cancer.

Group	Cancer	ID	Function	FDR *q*-value
down	BLCA	GO:0,036,297	interstrand cross-link repair	3.34E-03
up	BLCA	GO:0,010,729	positive regulation of hydrogen peroxide biosynthetic process	2.77E-05
down	BRCA	GO:0,048,284	organelle fusion	6.43E-03
up	BRCA	GO:0,040,030	regulation of molecular function, epigenetic	1.15E-10
down	CESC	GO:0,001,887	selenium compound metabolic process	2.47E-04
up	CESC	GO:0,048,846	axon extension involved in axon guidance	4.69E-03
down	CHOL	GO:0,034,587	piRNA metabolic process	1.32E-02
up	CHOL	GO:0,045,008	Depyrimidination	1.29E-12
down	COAD	GO:0,018,016	N-terminal peptidyl-proline dimethylation	4.41E-13
up	COAD	GO:0,034,214	protein hexamerization	1.23E-05
up	ESCA	GO:0,010,157	response to chlorate	4.36E-08
up	GBM	GO:2,001,162	positive regulation of histone H3-K79 methylation	7.71E-03
down	HNSC	GO:0,030,157	pancreatic juice secretion	2.35E-02
up	HNSC	GO:0,005,993	trehalose catabolic process	3.32E-03
down	KIRC	GO:0,046,597	negative regulation of viral entry into host cell	3.49E-18
down	KIRP	GO:0,007,144	female meiosis I	8.51E-03
up	KIRP	GO:0,046,878	positive regulation of saliva secretion	1.03E-02
down	LIHC	GO:0,045,008	Depyrimidination	2.81E-13
up	LIHC	GO:0,021,897	forebrain astrocyte development	1.48E-05
up	LUAD	GO:0,035,814	negative regulation of renal sodium excretion	3.03E-03
down	LUSC	GO:0,019,358	nicotinate nucleotide salvage	1.34E-03
up	LUSC	GO:0,045,006	DNA deamination	1.65E-02
down	PAAD	GO:0,070,585	protein localization to mitochondrion	1.63E-08
up	PAAD	GO:0,046,329	negative regulation of JNK cascade	1.04E-05
down	PCPG	GO:0,021,723	medullary reticular formation development	3.25E-05
up	PCPG	GO:0,006,335	DNA replication-dependent nucleosome assembly	1.72E-04
down	PRAD	GO:0,001,957	intramembranous ossification	5.15E-03
up	PRAD	GO:0,045,053	protein retention in Golgi apparatus	6.23E-09
down	READ	GO:0,060,123	regulation of growth hormone secretion	6.57E-05
up	READ	GO:0,042,742	defense response to bacterium	3.49E-04
down	SARC	GO:0,010,324	membrane invagination	7.69E-05
up	SKCM	GO:0,002,931	response to ischemia	8.74E-05
down	STAD	GO:0,080,184	response to phenylpropanoid	1.85E-04
down	THCA	GO:0,007,161	calcium-independent cell-matrix adhesion	8.69E-05
down	UCEC	GO:2,000,978	negative regulation of forebrain neuron differentiation	1.50E-03
up	UCEC	GO:0,070,208	protein heterotrimerization	1.10E-07

In this study, we hypothesized that up-methylated lncRNAs would exhibit decreased expression and vice versa. That is, there is a negative correlation between changes in methylation and expression levels. In order to screen out negative correlated lncRNAs in DMlncs, we used lncRNA expression data to identify differentially expressed lncRNAs in various cancers.

**FIGURE 5:**
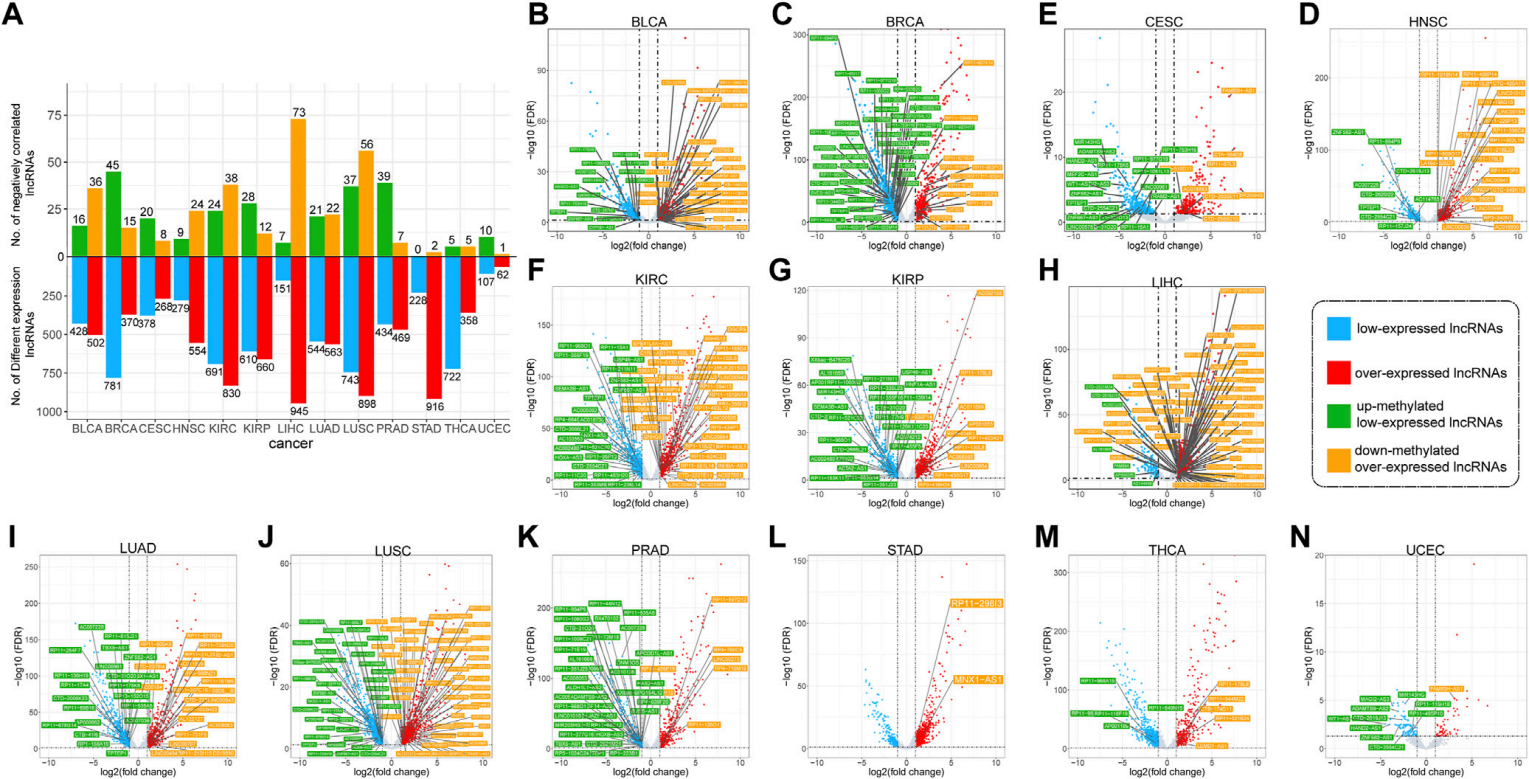
The negatively correlated lncRNAs for cancers **(A)** Quantitative analysis of lncRNAs that are differently expressed and negatively correlated **(B–N)** Distribution and names of negatively correlated lncRNAs for cancers. Red and blue dots represent differentially expressed lncRNAs. In which, blue represents down-expressed lncRNAs, and red represents up-expressed lncRNAs. Green and yellow boxes represent the names of negatively correlated lncRNAs. Where green indicates lncRNAs with up-methylated and low-expressed, and yellow represents lncRNAs with down-methylated and over-expressed.

LncRNA expression data of 13 cancer types were downloaded, and the number of differentially expressed lncRNAs in each cancer is shown in Supplementary Table S6. We identified 2,887 over-expressed lncRNAs and 2,375 low-expressed lncRNAs in different cancers. The total number of these lncRNAs was 4,155, and several genes were overlapped between two groups and displayed conflicting regulation patterns across different cancers. The numerical distribution of differentially expressed lncRNAs in various cancers is shown in Figure 5A. Although the number of over-expressed and low-expressed lncRNAs was similar in the majority of cancer types, there were significantly more low-expressed lncRNAs in BRCA and THCA, and significantly more over-expressed lncRNAs in LIHC and STAD.

Subsequently, negatively correlated lncRNAs (NClncs) were screened from differentially expressed lncRNAs. The number of NClncs in each cancer is shown in Figure 5A. NClncs were divided into two types: up-methylated-low-expressed lncRNAs (UMLElncs) and down-methylated-over-expressed lncRNAs (DMOElncs) based on the changes in expression and methylation levels. The two types of lncRNAs in each cancer are shown in Figure 5B–N. As seen from the figure, the number of NClncs was proportional to the number of differentially expressed lncRNAs. However, the proportion of differential lncRNAs was not balanced in several cancers. For example, STAD and THCA had many differentially expressed lncRNAs, but a small number of DMlncs, implying a small number of NClncs. The proportion of UMLElncs and DMOElncs was different in each cancer. For example, both types of lncRNAs for LUSC were abundant. HNSC and LIHC had significantly more DMOElncs, while BRCA and PRAD had significantly more UMLElncs.

The cancer-specific negatively correlated lncRNAs (CSNClncs) were then identified, and the results are shown in Table 4. Among the cancer-specific DMlncs, 49 NClncs were identified, with LIHC having the highest CSNClncs (11), followed by PRAD (7), BLCA (6) and KIRC (6). LUAD, THCA, and UCEC did not contain any CSNClnc.

**TABLE 4:** Cancer specific negatively correlated lncRNAs.

Cancer	Negatively correlated lncRNAs
BLCA	*XXbac-B476C20, RP5-943J3, RP11-390P2, AC006116, AC073046, RP11-514P8*
BRCA	*RP11-667K14, RP11-1094M14, RP11-497H17, LINC00619*
CESC	*MEF2C-AS1, CTA-384D8, CTD-2035E11*
HNSC	*LA16c-390E6, CTC-548K16, LA16c-325D7*
KIRC	*RP11-488L18, AC027601, RP1-118J21, HLA-F-AS1, SNHG12, EPB41L4A-AS1*
KIRP	*ACTA2-AS1, RP11-77H9, RP11-126K1*
LIHC	*AC007879, AC025335, CTC-246B18, HULC, LINC00665, RP11-215P8, RP11-890B15, RP11-968A15, RP11-973H7, RP3-395M20 and TEX41*
LUSC	*Z83851, RP11-311F12, RP11-757G1, RP11-12L8*
PRAD	*MIR205HG, LINC01018, JAZF1-AS1, RP4-639F20, RP1-223B1, RP11-597D13, LINC00115*
STAD	*MNX1-AS1, RP11-298I3*

Additionally, survival-correlated lncRNAs were identified by analyzing the CSNClncs of each cancer. As shown in Table 5, 29 lncRNAs were identified to associated with survival in ten cancers. These lncRNAs may be used as specifically diagnostic markers for corresponding cancers. They not only exhibited synergistic alterations in expression and methylation, but were also closely associated with the survival of cancer patients.

**TABLE 5:** Significantly survival associated lncRNAs in negatively correlated lncRNAs for each cancer.

Cancer	Count	lncRNAs
BLCA	5	*RP11-390P2, XXbac-B476C20, RP5-943J3, AC073046, AC006116*
BRCA	3	*LINC00619, RP11-497H17, RP11-667K14*
CESC	2	*MEF2C-AS1, CTD-2035E11*
HNSC	3	*LA16c-390E6, LA16c-325D7, CTC-548K16*
KIRC	5	*SNHG12, EPB41L4A-AS1, RP11-488L18, AC027601, RP1-118J21*
KIRP	2	*ACTA2-AS1, RP11-77H9*
LIHC	4	*RP11-215P8, RP11-968A15, RP11-973H7, CTC-246B18*
LUSC	2	*RP11-757G1, RP11-311F12*
PRAD	2	*RP1-223B1, LINC01018*
STAD	1	*MNX1-AS1*

**FIGURE 6:**
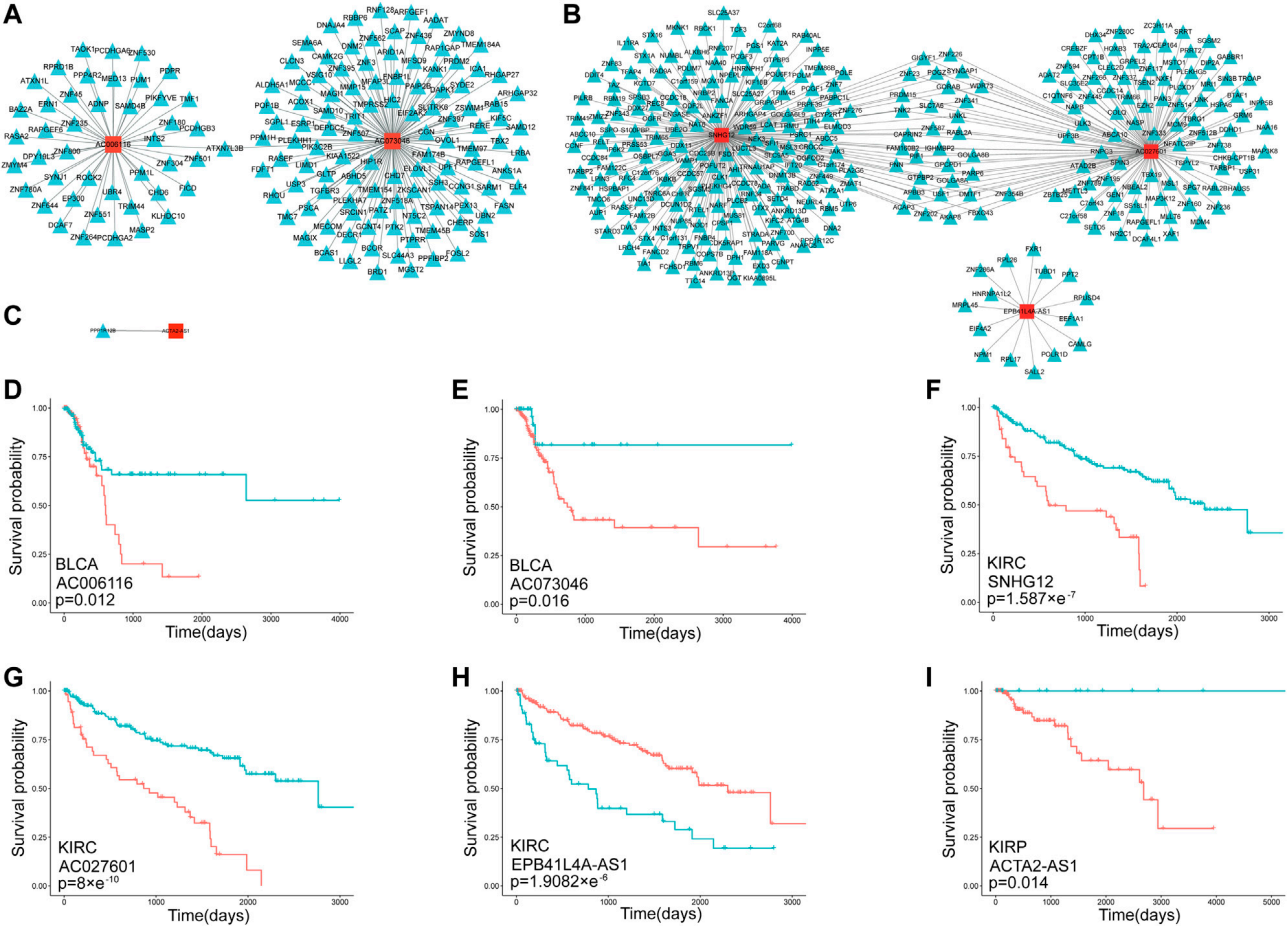
The negatively correlated lncRNAs which specific to each cancer in the ceRNA network **(A)**The ceRNAs of BLCA-specific negatively correlated lncRNAs **(B)** The ceRNAs of KIRC-specific negatively correlated lncRNAs **(C)** The ceRNAs of KIRP-specific negatively correlated lncRNAs **(D–I)** Kaplan-Meier curves for the lncRNAs associated with each cancer types. The red line represents the group with a high level of expression, while the blue line represents the group with a low level of expression. Additionally, the “+” on the lines represents patients who were lost to follow-up. At this point, the number of patients decreases but the overall survival rate remains stable.

The main function of lncRNAs was to combine miRNAs competing with mRNAs, thereby increasing mRNA expression. As the methylation of lncRNAs increased, their expression decreased, which indirectly led to the decrease in mRNA expression, and vice versa. Therefore, we screened the ceRNAs of DMlncs. First, the lncRNA-miRNA and mRNA-miRNA regulatory relationships were integrated. A lncRNA and a mRNA sharing more than two miRNAs were considered to have a ceRNA relationship. The correlation between lncRNA and mRNA expression was calculated for each cancer to confirm the ceRNA relationship. We maintained the ceRNA relationships that showed a positive correlation between lncRNA and mRNA expression. Each survival-related CSNClnc was put into the ceRNA network to search for associated mRNAs. The visualized results of ceRNAs for BLCA, KIRC and KIRP are shown in Figures 6A–C.

BLCA had two lncRNAs mapped into the ceRNA network (*AC006116* and *AC073046*). Both lncRNAs formed a ceRNA relationship with the mRNA *ATXN7L3B* and formed ceRNA relationships with some other mRNAs, respectively. *ATXN7L3B* have been confirmed to be associated with an increased risk of colorectal cancer (Leberfarb et al., 2020), and cytoplasmic *ATXN7L3B* interfered with the nuclear functions of the SAGA deubiquitinase module (Li et al., 2016). The SAGA complex was composed of two enzymatic modules, which house histone acetyltransferase (HAT) and deubiquitinase (DUB) activities. The DUB module was important for normal embryonic development (Glinsky, 2006; Lin et al., 2012), and alterations in the expression or structure of component proteins were linked to cancer (Lan et al., 2015). Therefore, *AC006116* and *AC073046*, as its ceRNAs, could regulate the expression of *ATXN7L3B* and were also closely related to cancer. The survival correlation of *AC006116* and *AC073046* in BLCA is shown in Figures 6D,E. Both lncRNAs were significantly associated with the survival of BLCA patients.

Three lncRNAs in KIRC were mapped into the ceRNA network, among which *SNHG12* and *AC027601* shared more than 30 mRNAs, while *EPB41L4A-AS1* did not share any mRNAs with the other two lncRNAs. The survival analysis results for the three lncRNAs in KIRC are shown in Figures 6F–H. The high expression of *SNHG12* and *AC027601* both showed a worse prognosis. However, the low expression group of *EPB41L4A-AS1* showed a worse prognosis. Therefore, we inferred that in KIRC, lncRNAs *SNHG12* and *AC027601* had a carcinogenic effect, whereas *EPB41L4A-AS1* had a tumor-suppressive effect, which explains why it shared no mRNAs with the other two lncRNAs. Numerous studies have established that *SNHG12* is associated with cancers (Zhang et al., 2020), and could be used as a potential therapeutic target and biomarker for human cancers (Tamang et al., 2019). DNA-methylation-mediated activation of *SNHG12* promoted temozolomide resistance in glioblastoma (Lu et al., 2020). *SNHG12* promoted tumor progression and sunitinib resistance by upregulating *CDCA3* in renal cell carcinoma (Liu et al., 2020). *EPB41L4A-AS1* was a repressor of the Warburg effect and played an important role in the metabolic reprogramming of cancer (Liao et al., 2019), and *EPB41L4A-AS1* has been identified as a potential biomarker in non-small cell lung cancer (Wang et al., 2020). At present, *AC027601* has been identified as a survival signature in renal clear cell carcinoma (Qi-Dong et al., 2020), but has not been reported in other cancers. Given that the three lncRNAs were simultaneously identified as KIRC-related lncRNAs in this study, we believed that *AC027601* should be closely associated with the occurrence and development of KIRC, and it is a newly identified cancer-related lncRNA.

Only *ACTA2-AS1* was mapped into the ceRNA network in KIRP, where it established a ceRNA relationship with *PPP1R12B*. *PPP1R12B* has been shown to inhibit tumor growth and metastasis by regulating Grb2/PI3K/Akt signaling in colorectal cancer (Ding et al., 2019). *ACTA2-AS1* plays an important role in a variety of cancers, for example, *ACTA2-AS1* is significantly associated with overall survival in ovarian cancer patients (Li and Zhan, 2019). *ACTA2-AS1* plays different roles in different cancers. *ACTA2-AS1* knockdown promotes liver cancer cell proliferation, migration and invasion (Zhou and Lv, 2019), while *ACTA2-AS1* suppresses lung adenocarcinoma progression (Ying et al., 2020), implying an inhibitory effect on the two cancers. However, *ACTA2-AS1* promotes cervical cancer progression (Luo et al., 2020), suggesting its carcinogenic role in cancer. Figure 6I shows the survival analysis result for *ACTA2-AS1* in KIRP. We believed that *ACTA2-AS1* had a carcinogenic effect in KIRP.

### Common lncRNA Biomarkers in Cancers

**FIGURE 7:**
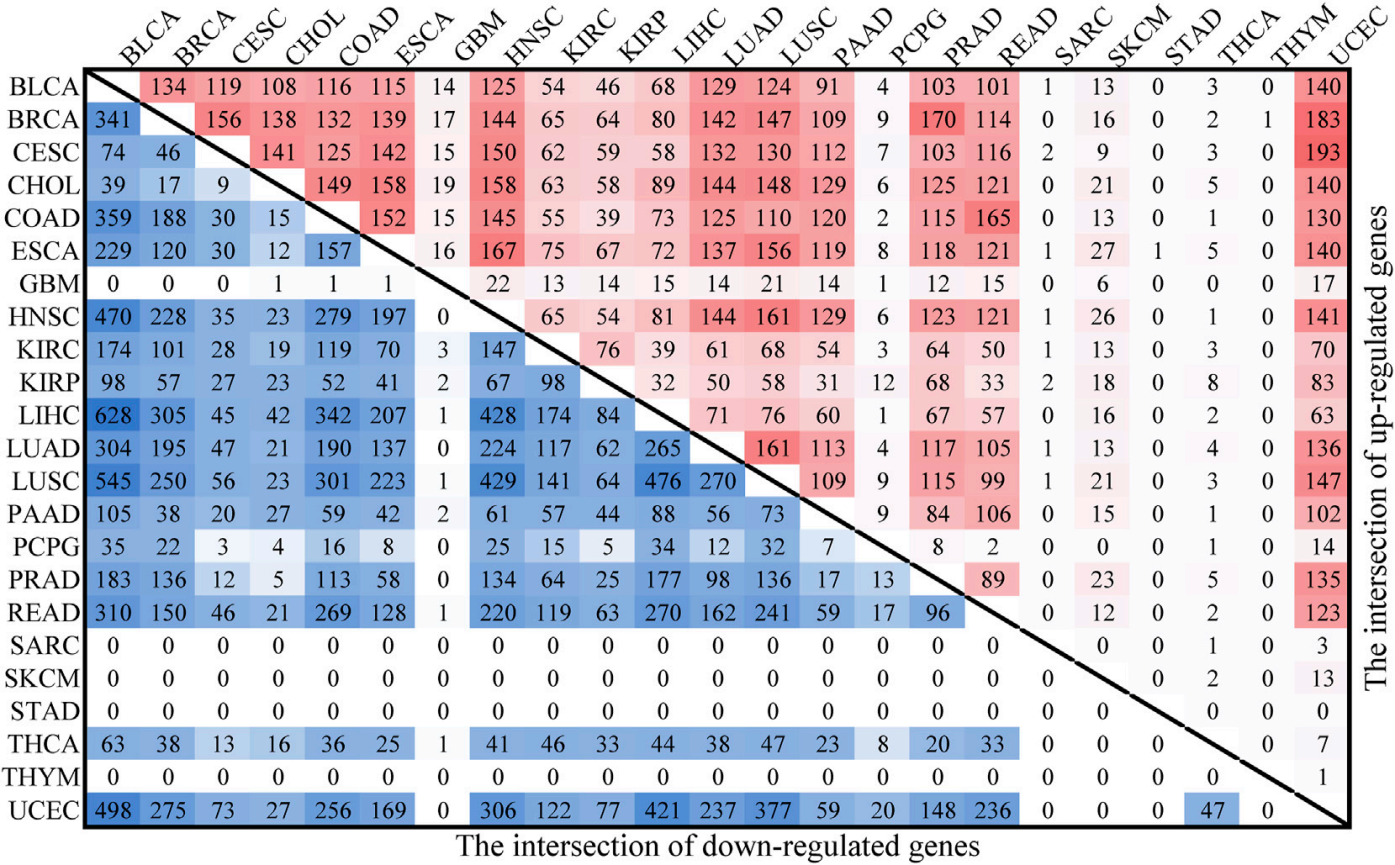
The intersection of DMlncs for cancers. The upper red triangle represents the intersection of up-methylated lncRNAs, while the lower blue triangle represents the intersection of down-methylated lncRNAs.

All cancer types exhibited infinite proliferation, transformation and ease of metastasis. Therefore, we sought to identify DMlncs common to different cancers to help understand the mechanisms underlying the occurrence of common features in cancers. First, the intersection of DMlncs were searched in cancers, and the results are shown in Figure 7. The upper triangle and lower triangle reflected the intersection of up-methylated lncRNAs and the intersection of down-methylated lncRNAs, respectively. The findings were consistent with the hypothesis that the larger the lncRNA set, the greater the overlap with other cancers. The intersections of DMlncs of SARC, STAD, and THYM with other cancers were small. The down-methylated lncRNAs of GBM and SKCM had small intersections with other cancers, whereas the up-methylated lncRNAs of THCA had small intersections with other cancers. There were amount of up-methylated lncRNAs in PCPG (62), but the overlaps with other cancers were small. Among the down-methylated lncRNAs, the intersection of KIRC and KIRP was the largest of KIRP, but only ranked 10th of the KIRC. Among the up-methylated lncRNAs, the intersection of KIRC and KIRP was the largest of KIRC, while was the second largest of KIRP.

**FIGURE 8:**
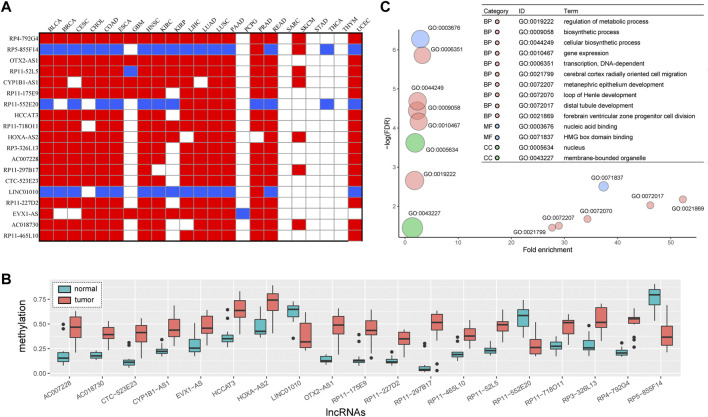
Common DMlncs of cancers **(A)** Methylation changes of common DMlncs in cancers. Red indicates up-methylation of the lncRNA, while blue indicates down-methylation in corresponding cancer **(B)** The functions of common DMlncs in cancer. Red represents a biological process (BP), blue represents a molecular function (MF), green represents cell component (CC), and the size of the bubble represents the amount of lncRNA enriched **(C)** A comparison of the methylation states of common DMlncs. Red represents tumor samples, while blue represents normal samples.

Subsequently, we extracted DMlncs which were common in various cancers. The findings indicated that there were 19 common DMlncs in more than 15 cancers (*RP4-792G4, RP5-855F14, OTX2-AS1, RP11-52L5, CYP1B1-AS1, RP11-175E9, RP11-552E20, HCCAT3, RP11-718O11, HOXA-AS2, RP3-326L13, AC007228, RP11-297B11, CTC-523E23, LINC01010, RP11-227D2, EVX1-AS, AC018730,* and *RP11-465L10*). Fourteen of them were up-methylated in all the cancers, indicating their carcinogenic potential, whereas three lncRNAs were down-methylated in the majority of cancers and may act as potential tumor suppressors (Figure 8A). Figure 8B shows the comparison of methylation levels of the 19 lncRNAs in tumor and normal samples. It is intuitive to conclude that there were significant differences in lncRNAs methylation levels between tumor and normal samples. Except for *LINC01010*, *RP11-552E20,* and *RP5-855F14*, the lncRNAs had a higher methylation level in tumor samples.

Among the 19 lncRNAs, O*XT2-AS1* was shown to be significantly down-methylated in lung squamous cell carcinoma and was closely associated with poor prognosis of cancer (Zheng et al., 2021). *CYP1B1-AS1* has been confirmed to play an important role in triple-negative breast cancer, lung adenocarcinoma and acute myeloid leukemia, and was associated with the prognosis of these cancers (Cheng et al., 2021; Ren et al., 2021; Vishnubalaji and Alajez, 2021). Abnormal methylation and low expression of *CTC-523E23* led to poor prognosis in patients with lung squamous cell carcinoma (Rui Li et al., 2020). Inhibition of *LINC01010* may promote the migration and invasion of lung cancer cells (Cao et al., 2020) and help in the prediction of neuroblastoma prognosis (Gao et al., 2020). *EVX1-AS* is closely associated with the prognosis of colon cancer and has been predicted to be potentially associated with the development of multiple cancers by LncRNADisease V2.0 (Bao et al., 2019; Gao et al., 2021). The findings revealed that the abnormalities of lncRNAs played an important role in the occurrence and development of cancer, and the unconfirmed lncRNAs could serve as entry points for future research. Additionally, numerous lncRNAs were identified as abnormal in lung cancer, indicating that lung cancer may be influenced by a variety of pathogenic factors.

Then, we investigated the functions of the 19 lncRNAs and performed functional enrichment analysis on these lncRNAs using the GREAT software. Figure 8C shows that these lncRNAs were enriched in processes required for organisms such as metabolism and biosynthesis, as well as those closely associated with the occurrence and development of cancer, such as gene expression and transcription.

NClncs in common lncRNAs were investigated in combination with the results of differentially expressed lncRNAs. Seven of the 19 lncRNAs were found to be differentially expressed, and four (*CYP1B1-AS1* (BLCA, BRCA, and LUSC), *AC007228* (BLCA, HNSC, LIHC, LUAD, LUSC and PRAD)*, HOXA-AS2* (BRCA) and *LINC01010* (HNSC)) of them had a negative correlation in multiple cancers. Additionally, we analyzed the four NClncs shared by cancers for their correlation with survival, and survival-related lncRNAs were identified. The four lncRNAs were associated with patients’ survival in a variety of cancers, including *CYP1B1-AS1* in eight types of cancers (BRCA, HNSC, KIRC, KIRP, LIHC, PUAD, PRAD and STAD), *AC007228* in seven types of cancers (BLCA, HNSC, KIRC, KIRP, LIHC, LUAD and THCA), *LINC01010* in five types of cancers (BRCA, LIHC, LUAD, LUSC and THCA) and *HOXA-AS2* in four types of cancers (BRCA, KIRC, KIRP and THCA) (Supplementary Figures S1–S4).

**FIGURE 9:**
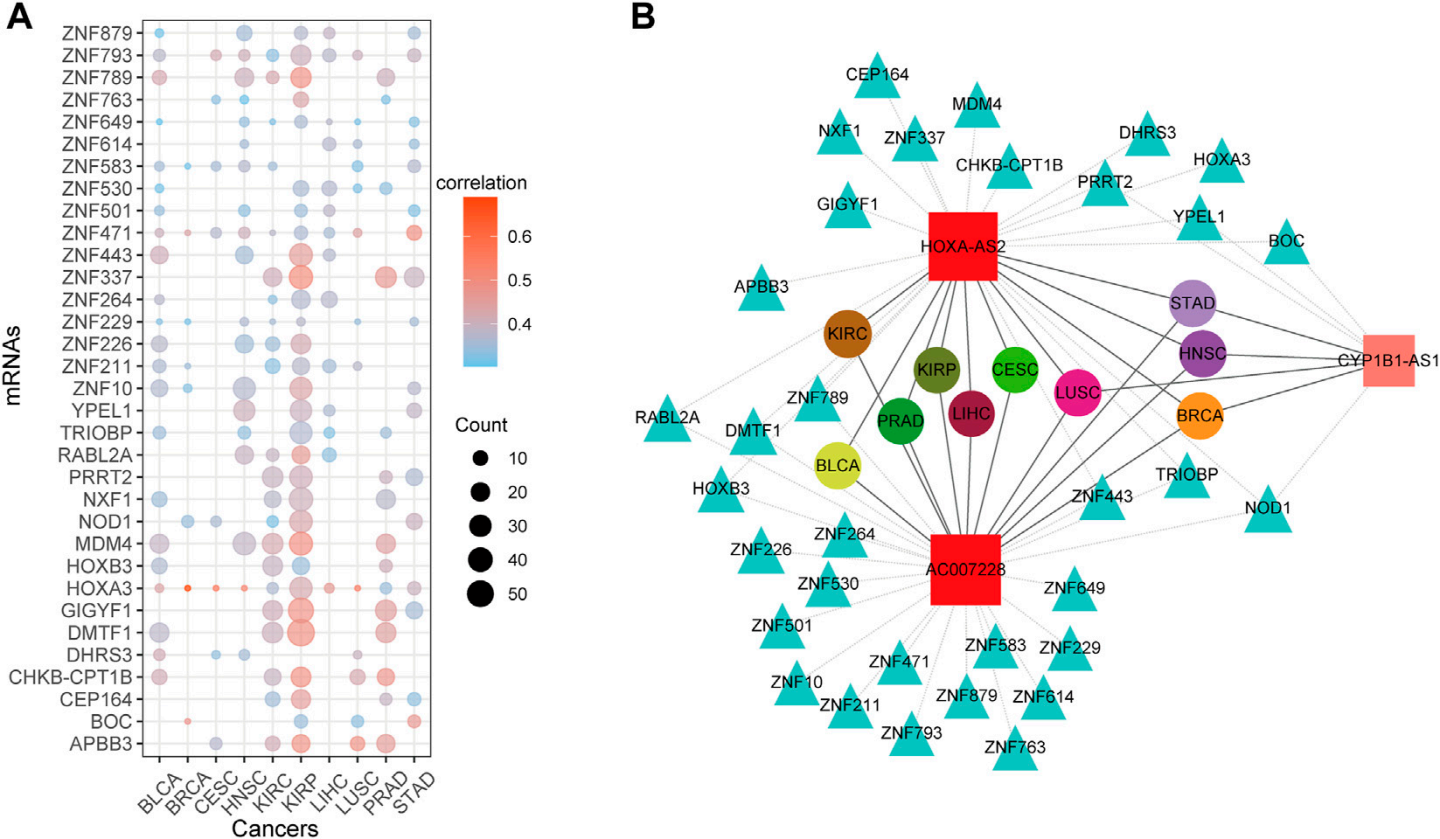
The ceRNA network of common DMlncs of cancers **(A)** The ceRNA distribution of mRNAs in each cancer type. The color of the dots represents the mean of the correlation coefficient for the lncRNAs that form ceRNA relationships in corresponding cancer. The size of the dots represents the number of lncRNAs that have a ceRNA relationship with the corresponding mRNA in cancer **(B)** The ceRNA network. Square nodes represent lncRNAs, triangle nodes represent mRNAs, and round nodes represent cancer types. The size of lncRNA nodes is proportional to the number of cancer types sharing it, and nodes of different cancer types are distinguished by different colors.

To further validate the cancer-common lncRNAs identified in this study, significantly survival-related lncRNAs were mapped to the ceRNA network, and only mRNAs shared by more than four types of cancers were selected for further study. Finally, a subnetwork comprising 33 mRNAs and three lncRNAs (*AC007228, CYP1B1-AS1,* and *HOXA-AS2*) was identified in ten cancers. The ceRNA distribution of the 33 mRNAs in cancers is shown in Figure 9A. Although these mRNAs were shared by multiple cancers, they generally formed ceRNA relationships with a greater number of lncRNAs and had higher correlation coefficients in KIRP. *HOXA3* had ceRNA lncRNAs in ten cancers and showed high correlation coefficients in BRCA, CESC, HNSC and LUSC. Figure 9B shows the ceRNA subnetwork, in which *HOXA-AS2* and *AC007228* were shared by ten cancers, and the two lncRNAs shared some ceRNA relationships with some mRNAs (*DMTF1, HOXB3, NOD1, RABL2A, TRIOBP, ZNF443,* and *ZNF789*) but formed ceRNA relationships with some other mRNAs, respectively (*AC007228: ZNF10, ZNF211, ZNF229, ZNF471, ZNF583, ZNF614, ZNF649, ZNF763, ZNF793,* and *ZNF879*; *HOXA-AS2*: *DHRS3* and *HOXA3*).

**FIGURE 10:**
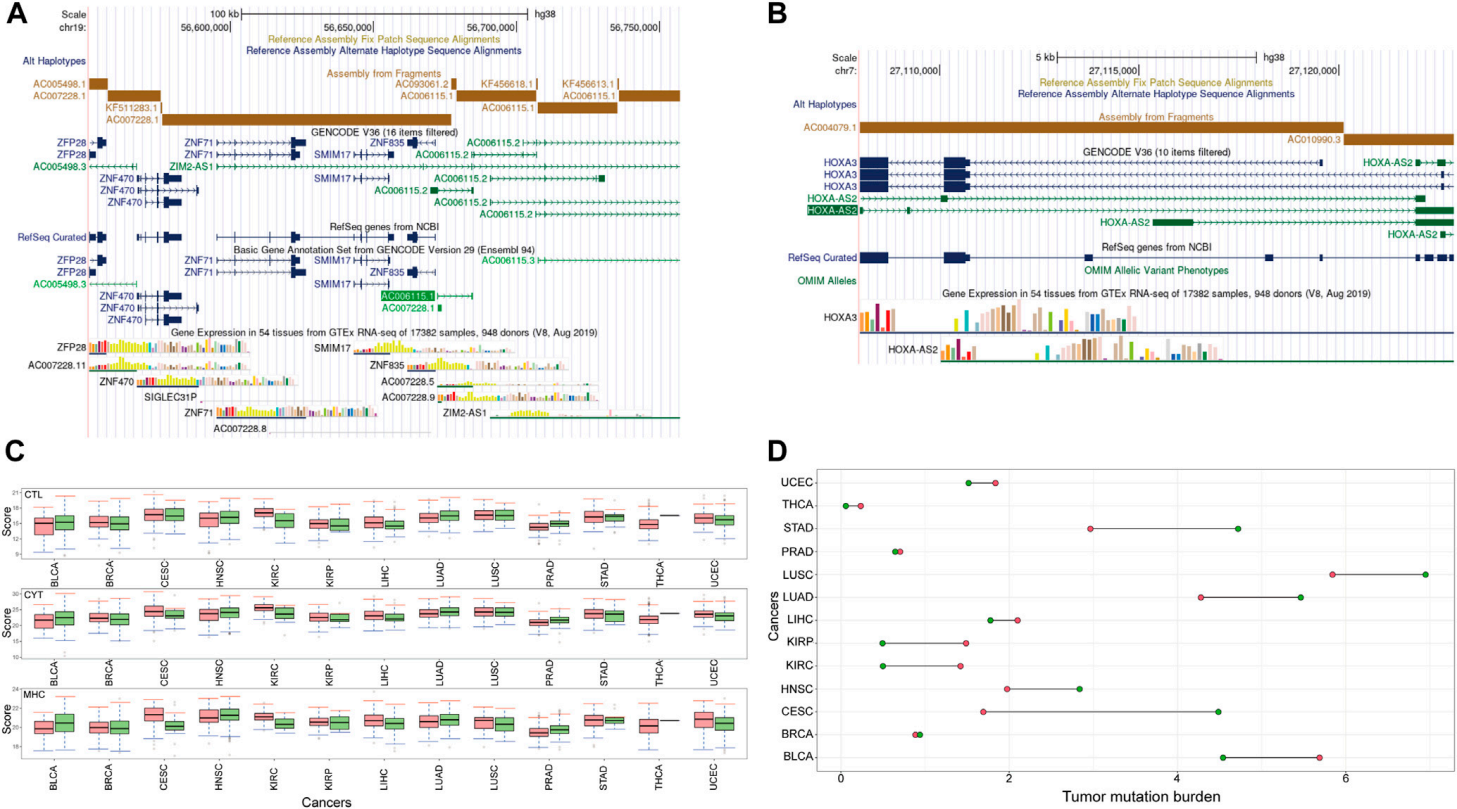
The characteristics of *HOXA-AS2* and *AC007228* **(A)** The genomic location of *AC007228* **(B)** The genomic location of *HOXA-AS2* **(C)** Immune response scores of *HOXA-AS2* and *AC007228* **(D)** TMB of *HOXA-AS2* and *AC007228.*

The genomic locations of *HOXA-AS2* and *AC007228* were checked. As shown in Figure 10A, the genomic locations of lncRNAs belonging to the *AC007228* family and mRNAs belonging to the *ZNF* family were extremely similar. As shown in Figure 10B, *HOXA-AS2* (Zhao et al., 2013) and its ceRNA *HOXA3* were both located in the same genomic region. Their sequences were similar, and the ceRNA relationships were generated by the lncRNAs’ cis-regulatory interactions with mRNAs.

The major histocompatibility complex (MHC) region was one of the regions with the highest gene density and polymorphism. High-throughput sequencing and other technologies confirmed the role of MHC in disease and showed that MHC was associated with cancer and neurological diseases in addition to infection and autoimmune diseases. MHC is involved in antigen recognition during the immune response and is capable of inducing immune cells to participate in immune response (Trowsdale and Knight, 2013). Studies have also reported that immune cytolytic activity (CYT) is positively correlated with the presence of inhibitory receptors (*PDCD1, PDL1, CTLA4, LAG3, TIM3,* and *IDO1*), and the presence of CYT is more responsive to immune checkpoint inhibition, suggesting that it can be used as a key marker for immune checkpoint therapy (Narayanan et al., 2018; Wang et al., 2019). Immune control of tumor lesions requires local antigen recognition, activation and amplification of tumor-specific cytotoxic T lymphocytes (CTL). The activated CTL infiltrate the tumor microenvironment and scan the tumor tissue, where they directly interact with the target cells, inducing tumor cell apoptosis and atrophy (Basu et al., 2016). First, effector T lymphocytes are required to migrate to the tumor foci, a process referred to as immune cell infiltration. Following that, they have to make physical contact with the tumor cells and scan their MHC. Finally, by releasing perforin or fas/fasl to bind target cells, CTL activates and induces apoptosis (Weigelin et al., 2011). Therefore, we assess the immunological effects of *HOXA-AS2* and *AC007228* using MHC, CYT, and CTL scores.

To improve the evaluation of the effect of lncRNAs on immunity, the R package “ConsensusClusterPlus” (Wilkerson and Hayes, 2010) was used to cluster samples of each cancer based on their expression profiles of *HOXA-AS2* and *AC007228*. We varied the parameter k from two to six, and then selected the optimal subtypes for subsequent immune score evaluation. The score comparison shown in Figure 10C demonstrates that all three scores were consistent across cancer subtypes, indicating that the identified key lncRNAs may aid in predicting the immunological status of cancer patients and provide a basis for tumor treatment. Subsequently, the Wilcoxon rank-sum test was used to compare the immunity scores of different subtypes, and significant differences were observed in the immunity scores of different subtypes in KIRC, LIHC, LUAD, and PRAD.

Finally, we assessed the tumor mutation burden (TMB) among subtypes. TMB was a novel biological target for which therapeutic impact may be predicted. Previous research has demonstrated that the more somatic mutations a cancer patient possesses, the more likely it is that new antigens are produced. Antigen peptides could be loaded onto the MHC and displayed on the cell surface, aiding in their recognition by T cells (Jiang et al., 2018). Therefore, cancer patients with high TMB levels responded better to immune checkpoint blockade therapy. As shown in Figure 10D, the TMB of KIRC, KIRP, LIHC, LUAD, and UCEC corresponded to the immunity score. Subtypes of key lncRNAs played an important role in the immune effect in KIRC, LIHC, and LUAD.

## Discussion

LncRNAs have been implicated in the occurrence and development of cancer. This study aimed to identify the specific and common lncRNAs with aberrant methylation in pan-cancer. After searching for DMlncs in a variety of cancers, the pan-cancer results were compared. Subsequently, data on lncRNA expression, lncRNA methylation and mRNA expression was integrated to identify lncRNAs with a negative correlation between methylation and expression changes, and survival analysis was performed to further verify the results. Following that, survival-related lncRNAs were mapped to the ceRNA network, and pan-cancer biomarkers were identified by examining the connection characteristics of the network. Finally, the immune effect of the lncRNAs was verified.

In this study, DMlncs for 23 cancers were acquired, and cancer-specific lncRNAs and cancer-common lncRNAs were identified. The NClncs were further screened for ten cancers, and the correlation between the lncRNAs and mRNAs, as well as the association with survival were verified. Cancer-specific lncRNAs may be used as diagnostic biomarkers for corresponding cancers. In clinical application, these lncRNAs could apply to make detection kits of corresponding cancers. Common lncRNAs in pan-cancers could be used to understand the mechanism underlying common features in cancers. These lncRNAs can be used for the development of targeting drugs for the remission of general symptoms and treatment of cancer.

This study yielded significant results. Not only did we validate several previously known cancer-associated lncRNAs, but we also identified new cancer-related lncRNAs. *AC027601* was identified as a novel KIRC-associated lncRNA, and *ACTA2-AS1* was discovered to be carcinogenic in KIRP. Additionally, two lncRNAs, *HOXA-AS2,* and *AC007228* were identified as pan-cancer lncRNAs.

However, there are some limitations to this study. Because the number of DMlncs for SARC, STAD, and THYM was less than ten, the more systematic comparisons for these cancers were impossible. The number of normal samples with methylation data for these three cancers was less than ten. The sample proportion was skewed when differences were calculated, resulting in less statistically significant results. Additionally, the DNA methylation data only included 450k arrays and did not cover the entire genome, which might have contributed to the study’s insufficiency outcomes. We only identified the epigenetically dysregulated lncRNAs based on DNA methylation, although N6-methyladenosine (m6A) as the RNA post-transcriptional modification has been shown to influence the function of RNAs as well. We did not evaluate the relationship between m6A and lncRNA due to the lack of data.

In the future, more complete data sets on cancers may be collected to allow for more rigorous comparisons. We may use copy number data to analyze the change in copy number of lncRNA and conduct a more comprehensive investigation of the change and function of lncRNA in cancer. Additionally, other types of omics data may be integrated, and factors affecting lncRNAs and gene expression could be evaluated more comprehensively bringing the research process closer to the way molecules interact in the human body. With the development of sequencing techniques, additional methylation data sets such as HM850K, whole-genome bisulfite sequencing (WGBS), and reduced representation bisulfite sequencing (RRBS), as well as other types of methylation data sets, will be used to analyze the function of DNA methylation for lncRNAs.

In general, this study screened cancer-related lncRNA biomarkers based on their methylation alterations and their competing mRNAs. This study considered multiple omics data more comprehensively and used more stringent screening criteria, which effectively eliminated of data deviation errors. The lncRNA biomarkers identified in this study may aid in the investigation of cancer mechanisms.

## Data Availability

Publicly available datasets were analyzed in this study. This data can be found here: The datasets ANALYZED for this study can be found in the TCGA (https://portal.gdc.cancer.gov/) and TANRIC (https://www.tanric.org/).

## Glossary

BLCA: Bladder Urothelial Carcinoma
BRCA: Breast Invasive Carcinoma
CESC: Cervical Squamous Cell Carcinoma and Endocervical Adenocarcinoma
CHOL: Cholangiocarcinoma
COAD: Colon Adenocarcinoma
ESCA: Esophageal Carcinoma
GBM: Glioblastoma Multiforme
HNSC: Head and Neck Squamous Cell Carcinoma
KIRC: Kidney Renal Clear Cell Carcinoma
KIRP: Kidney Renal Papillary Cell Carcinoma
LIHC: Liver Hepatocellular Carcinoma
LUAD: Lung Adenocarcinoma
LUSC: Lung Squamous Cell Carcinoma
PAAD: Pancreatic Adenocarcinoma
PCPG: Pheochromocytoma and Paraganglioma
PRAD: Prostate Adenocarcinoma
READ: Rectum Adenocarcinoma
SARC: Sarcoma
SKCM: Skin Cutaneous Melanoma
STAD: Stomach Adenocarcinoma
THCA: Thyroid Carcinoma
THYM: Thymoma
UCEC: Uterine Corpus Endometrial Carcinoma
lncRNA: Long non-coding RNAs
DMlnc: differentially methylated lncRNA
NClnc: negatively correlated lncRNA
UMLElnc: up-methylated-low-expressed lncRNA
DMOElnc: down-methylated-over-expressed lncRNA
CSNClncs: cancer-specific negatively correlated lncRNAs
MHC: major histocompatibility complex
CYT: immunolytic cell activity
CTL: cytotoxic T cell
TMB: tumor mutation burden
lncRNA: Long non-coding RNA
DMlnc: Differentially Methylated lncRNA
NClnc: Negatively Correlated lncRNA
UMLElnc: Up-Methylated-Low-Expressed lncRNA
DMOElnc: Down-Methylated-Over-Expressed lncRNA
CSNClnc: Cancer-Specific Negatively Correlated lncRNA
MHC: Major Histocompatibility Complex
CYT: Cytolytic Activity
CTL: Cytotoxic T Lymphocyte
TMB: Tumor Mutation Burden

## References

[B1] Aryee M. J. Jaffe A. E. Corrada-Bravo H. Ladd-Acosta C. Feinberg A. P. Hansen K. D. (2014). Minfi: a Flexible and Comprehensive Bioconductor Package for the Analysis of Infinium DNA Methylation Microarrays. Bioinformatics 30 (10), 1363–1369. 10.1093/bioinformatics/btu049 24478339 PMC4016708

[B2] Bao Z. Yang Z. Huang Z. Zhou Y. Cui Q. Dong D. (2019). LncRNADisease 2.0: an Updated Database of Long Non-coding RNA-Associated Diseases. Nucleic Acids Res. 47 (D1), D1034–D1037. 10.1093/nar/gky905 30285109 PMC6324086

[B74] Bao S. Zhao H. Yuan J. Fan D. Zhang Z. Su J. (2020). Computational Identification of Mutator-Derived Lncrna Signatures of Genome Instability For Improving the Clinical Outcome of Cancers: A Case Study in Breast Cancer. Brief Bioinform 21 (5), 1742–1755. 10.1093/bib/bbz118 31665214

[B3] Bao G. Xu R. Wang X. Ji J. Wang L. Li W. (2021). Identification of lncRNA Signature Associated with Pan-Cancer Prognosis. IEEE J. Biomed. Health Inf. 25 (6), 2317–2328. 10.1109/JBHI.2020.3027680 32991297

[B4] Bartonicek N. Maag J. L. V. Dinger M. E. (2016). Long Noncoding RNAs in Cancer: Mechanisms of Action and Technological Advancements. Mol. Cancer 15 (1), 43. 10.1186/s12943-016-0530-6 27233618 PMC4884374

[B5] Basu R. Whitlock B. M. Husson J. Le Floc’h A. Jin W. Oyler-Yaniv A. (2016). Cytotoxic T Cells Use Mechanical Force to Potentiate Target Cell Killing. Cell 165 (1), 100–110. 10.1016/j.cell.2016.01.021 26924577 PMC4808403

[B6] Cabili M. N. Trapnell C. Goff L. Koziol M. Tazon-Vega B. Regev A. (2011). Integrative Annotation of Human Large Intergenic Noncoding RNAs Reveals Global Properties and Specific Subclasses. Genes Dev. 25 (18), 1915–1927. 10.1101/gad.17446611 21890647 PMC3185964

[B7] Cao Q. Dong Z. Liu S. An G. Yan B. Lei L. (2020). Construction of a Metastasis-Associated ceRNA Network Reveals a Prognostic Signature in Lung Cancer. Cancer Cell Int. 20, 208. 10.1186/s12935-020-01295-8 32518519 PMC7271455

[B8] Chen Y. G. Satpathy A. T. Chang H. Y. (2017). Gene Regulation in the Immune System by Long Noncoding RNAs. Nat. Immunol. 18 (9), 962–972. 10.1038/ni.3771 28829444 PMC9830650

[B9] Chen J. Yu Y. Li H. Hu Q. Chen X. He Y. (2019). Long Non-coding RNA PVT1 Promotes Tumor Progression by Regulating the miR-143/HK2 axis in Gallbladder Cancer. Mol. Cancer 18 (1), 33. 10.1186/s12943-019-0947-9 30825877 PMC6397746

[B10] Cheng Y. Wang X. Qi P. Liu C. Wang S. Wan Q. (2021). Tumor Microenvironmental Competitive Endogenous RNA Network and Immune Cells Act as Robust Prognostic Predictor of Acute Myeloid Leukemia. Front. Oncol. 11, 584884. 10.3389/fonc.2021.584884 33898304 PMC8063692

[B11] Ding C. Tang W. Wu H. Fan X. Luo J. Feng J. (2019). The PEAK1-Ppp1r12b axis Inhibits Tumor Growth and Metastasis by Regulating Grb2/PI3K/Akt Signalling in Colorectal Cancer. Cancer Lett. 442, 383–395. 10.1016/j.canlet.2018.11.014 30472186

[B76] Dong Y. Xiao Y. Shi Q. Jiang C. (2019). Dysregulated lncRNA-miRNA-mRNA Network Reveals Patient Survival-Associated Modules and RNA Binding Proteins in Invasive Breast Carcinoma. Front. Genet. 10, 1284. 10.3389/fgene.2019.01284 32010179 PMC6975227

[B12] Esposito R. Bosch N. Lanzós A. Polidori T. Pulido-Quetglas C. Johnson R. (2019). Hacking the Cancer Genome: Profiling Therapeutically Actionable Long Non-coding RNAs Using CRISPR-Cas9 Screening. Cancer Cell 35 (4), 545–557. 10.1016/j.ccell.2019.01.019 30827888

[B13] Gao L. Lin P. Chen P. Gao R. Z. Yang H. He Y. (2020). A Novel Risk Signature that Combines 10 Long Noncoding RNAs to Predict Neuroblastoma Prognosis. J. Cell Physiol. 235 (4), 3823–3834. 10.1002/jcp.29277 31612488

[B14] Gao M. Guo Y. Xiao Y. Shang X. (2021). Comprehensive Analyses of Correlation and Survival Reveal Informative lncRNA Prognostic Signatures in Colon Cancer. World J. Surg. Onc. 19 (1), 104. 10.1186/s12957-021-02196-4 PMC8035745 33836755

[B15] Glinsky G. V. (2006). Genomic Models of Metastatic Cancer: Functional Analysis of Death-From-Cancer Signature Genes Reveals Aneuploid, Anoikis-Resistant, Metastasis-Enabling Phenotype with Altered Cell Cycle Control and Activated PcG Protein Chromatin Silencing Pathway. Cell Cycle 5 (11), 1208–1216. 10.4161/cc.5.11.2796 16760651

[B73] Hou P. Bao S. Fan D. Yan C. Su J. Qu J. (2021). Machine Learning-Based Integrative Analysis of Methylome and Transcriptome Identifies Novel Prognostic DNA Methylation Signature in Uveal Melanoma. Brief Bioinform. 22 (4). 10.1093/bib/bbaa371 33367533

[B16] Huarte M. (2015). The Emerging Role of lncRNAs in Cancer. Nat. Med. 21 (11), 1253–1261. 10.1038/nm.3981 26540387

[B17] Jiang P. Gu S. Pan D. Fu J. Sahu A. Hu X. (2018). Signatures of T Cell Dysfunction and Exclusion Predict Cancer Immunotherapy Response. Nat. Med. 24 (10), 1550–1558. 10.1038/s41591-018-0136-1 30127393 PMC6487502

[B18] Lan X. Koutelou E. Schibler A. C. Chen Y. C. Grant P. A. Dent S. Y. R. (2015). Poly(Q) Expansions in ATXN7 Affect Solubility but Not Activity of the SAGA Deubiquitinating Module. Mol. Cell Biol. 35 (10), 1777–1787. 10.1128/MCB.01454-14 25755283 PMC4405643

[B19] Lauss M. Donia M. Harbst K. Andersen R. Mitra S. Rosengren F. (2017). Mutational and Putative Neoantigen Load Predict Clinical Benefit of Adoptive T Cell Therapy in Melanoma. Nat. Commun. 8 (1), 1738. 10.1038/s41467-017-01460-0 29170503 PMC5701046

[B20] Leberfarb E. Y. Degtyareva A. O. Brusentsov II Maximov V. N. Voevoda M. I. Autenshlus A. I. (2020). Potential Regulatory SNPs in the ATXN7L3B and KRT15 Genes Are Associated with Gender-specific Colorectal Cancer Risk. Pers. Med. 17 (1), 43–54. 10.2217/pme-2019-0059 31797724

[B21] Li N. Zhan X. (2019). Identification of Clinical Trait-Related lncRNA and mRNA Biomarkers with Weighted Gene Co-expression Network Analysis as Useful Tool for Personalized Medicine in Ovarian Cancer. EPMA J. 10 (3), 273–290. 10.1007/s13167-019-00175-0 31462944 PMC6695468

[B22] Li X.-Q. Guo Y. Y. De W. (2012). DNA Methylation and microRNAs in Cancer. Wjg 18 (9), 882–888. 10.3748/wjg.v18.i9.882 22408346 PMC3297046

[B72] Li Y. Xu J. Chen H. Zhao Z. Li S. Bai J. (2013). Characterizing Genes With Distinct Methylation Patterns in the Context of Protein-Protein Interaction Network: Application to Human Brain Tissues. PLoS One 8 (6), e65871 . 10.1371/journal.pone.0065871 23776563 PMC3680465

[B23] Li J.-H. Liu S. Zhou H. Qu L.-H. Yang J.-H. (2014). starBase v2.0: Decoding miRNA-ceRNA, miRNA-ncRNA and Protein-RNA Interaction Networks from Large-Scale CLIP-Seq Data. Nucl. Acids Res. 42 (Database issue), D92–D97. 10.1093/nar/gkt1248 24297251 PMC3964941

[B24] Li J. Han L. Roebuck P. Diao L. Liu L. Yuan Y. (2015). TANRIC: An Interactive Open Platform to Explore the Function of lncRNAs in Cancer. Cancer Res. 75 (18), 3728–3737. 10.1158/0008-5472.CAN-15-0273 26208906 PMC4573884

[B25] Li W. Atanassov B. S. Lan X. Mohan R. D. Swanson S. K. Farria A. T. (2016). Cytoplasmic ATXN7L3B Interferes with Nuclear Functions of the SAGA Deubiquitinase Module. Mol. Cell Biol. 36 (22), 2855–2866. 10.1128/MCB.00193-16 27601583 PMC5086525

[B26] Liao M. Liao W. Xu N. Li B. Liu F. Zhang S. (2019). LncRNA EPB41L4A-AS1 Regulates Glycolysis and Glutaminolysis by Mediating Nucleolar Translocation of HDAC2. EBioMedicine 41, 200–213. 10.1016/j.ebiom.2019.01.035 30796006 PMC6444057

[B27] Lin C. Yang L. (2018). Long Noncoding RNA in Cancer: Wiring Signaling Circuitry. Trends Cell Biol. 28 (4), 287–301. 10.1016/j.tcb.2017.11.008 29274663 PMC5869122

[B28] Lin Z. Yang H. Kong Q. Li J. Lee S.-M. Gao B. (2012). USP22 Antagonizes P53 Transcriptional Activation by Deubiquitinating Sirt1 to Suppress Cell Apoptosis and Is Required for Mouse Embryonic Development. Mol. Cell 46 (4), 484–494. 10.1016/j.molcel.2012.03.024 22542455

[B29] Liu Y. Cheng G. Huang Z. Bao L. Liu J. Wang C. (2020). Long Noncoding RNA SNHG12 Promotes Tumour Progression and Sunitinib Resistance by Upregulating CDCA3 in Renal Cell Carcinoma. Cell Death Dis. 11 (7), 515. 10.1038/s41419-020-2713-8 32641718 PMC7343829

[B30] Lu C. Wei Y. Wang X. Zhang Z. Yin J. Li W. (2020). DNA-methylation-mediated Activating of lncRNA SNHG12 Promotes Temozolomide Resistance in Glioblastoma. Mol. Cancer 19 (1), 28. 10.1186/s12943-020-1137-5 32039732 PMC7011291

[B31] Luo L. Wang M. Li X. Luo C. Tan S. Yin S. (2020). A Novel Mechanism by Which ACTA2-AS1 Promotes Cervical Cancer Progression: Acting as a ceRNA of miR-143-3p to Regulate SMAD3 Expression. Cancer Cell Int. 20, 372. 10.1186/s12935-020-01471-w 32774166 PMC7409411

[B32] Martens-Uzunova E. S. Böttcher R. Croce C. M. Jenster G. Visakorpi T. Calin G. A. (2014). Long Noncoding RNA in Prostate, Bladder, and Kidney Cancer. Eur. Urol. 65 (6), 1140–1151. 10.1016/j.eururo.2013.12.003 24373479

[B33] Martín-Subero J. I. (2011). How Epigenomics Brings Phenotype into Being. Pediatr. Endocrinol. Rev. 9 Suppl 1, 506–510. 22423506

[B34] Marwitz S. Scheufele S. Perner S. Reck M. Ammerpohl O. Goldmann T. (2017). Epigenetic Modifications of the Immune-Checkpoint Genes CTLA4 and PDCD1 in Non-small Cell Lung Cancer Results in Increased Expression. Clin. Epigenet. 9, 51. 10.1186/s13148-017-0354-2 PMC5426039 28503213

[B35] McLean C. Y. Bristor D. Hiller M. Clarke S. L. Schaar B. T. Lowe C. B. (2010). GREAT Improves Functional Interpretation of Cis-Regulatory Regions. Nat. Biotechnol. 28 (5), 495–501. 10.1038/nbt.1630 20436461 PMC4840234

[B36] Mortazavi A. Williams B. A. McCue K. Schaeffer L. Wold B. (2008). Mapping and Quantifying Mammalian Transcriptomes by RNA-Seq. Nat. Methods 5 (7), 621–628. 10.1038/nmeth.1226 18516045 PMC13303166

[B37] Narayanan S. Kawaguchi T. Yan L. Peng X. Qi Q. Takabe K. (2018). Cytolytic Activity Score to Assess Anticancer Immunity in Colorectal Cancer. Ann. Surg. Oncol. 25 (8), 2323–2331. 10.1245/s10434-018-6506-6 29770915 PMC6237091

[B38] Neri F. Rapelli S. Krepelova A. Incarnato D. Parlato C. Basile G. (2017). Intragenic DNA Methylation Prevents Spurious Transcription Initiation. Nature 543 (7643), 72–77. 10.1038/nature21373 28225755

[B39] Pauken K. E. Sammons M. A. Odorizzi P. M. Manne S. Godec J. Khan O. (2016). Epigenetic Stability of Exhausted T Cells Limits Durability of Reinvigoration by PD-1 Blockade. Science 354 (6316), 1160–1165. 10.1126/science.aaf2807 27789795 PMC5484795

[B40] PCAWG Transcriptome Core Group Calabrese C. Davidson N. R. Demircioğlu D. Fonseca N. A. He Y. (2020). Genomic Basis for RNA Alterations in Cancer. Nature 578 (7793), 129–136. 10.1038/s41586-020-1970-0 32025019 PMC7054216

[B41] Qi-Dong X. Yang X. Lu J.-L. Liu C.-Q. Sun J.-X. Li C. (2020). Development and Validation of a Nine-Redox-Related Long Noncoding RNA Signature in Renal Clear Cell Carcinoma. Oxid. Med. Cell. Longev. 2020, 1–30. 10.1155/2020/6634247 PMC7781722 33425212

[B42] Ren J. Wang A. Liu J. Yuan Q. (2021). Identification and Validation of a Novel Redox-Related lncRNA Prognostic Signature in Lung Adenocarcinoma. Bioengineered 12 (1), 4331–4348. 10.1080/21655979.2021.1951522 34338158 PMC8806475

[B43] Ritchie M. E. Phipson B. Wu D. Hu Y. Law C. W. Shi W. (2015). Limma Powers Differential Expression Analyses for RNA-Sequencing and Microarray Studies. Nucleic Acids Res. 43 (7), e47. 10.1093/nar/gkv007 25605792 PMC4402510

[B44] Rooney M. S. Shukla S. A. Wu C. J. Getz G. Hacohen N. (2015). Molecular and Genetic Properties of Tumors Associated with Local Immune Cytolytic Activity. Cell 160 (1-2), 48–61. 10.1016/j.cell.2014.12.033 25594174 PMC4856474

[B45] Li R. Yin Y.-H. Jin J. Liu X. Zhang M.-Y. Yang Y.-E. (2020). Integrative Analysis of DNA Methylation-Driven Genes for the Prognosis of Lung Squamous Cell Carcinoma Using MethylMix. Int. J. Med. Sci. 17 (6), 773–786. 10.7150/ijms.43272 32218699 PMC7085273

[B46] Saghafinia S. Mina M. Riggi N. Hanahan D. Ciriello G. (2018). Pan-Cancer Landscape of Aberrant DNA Methylation across Human Tumors. Cell Rep. 25 (4), 1066–1080.e8. 10.1016/j.celrep.2018.09.082 30355485

[B47] Sen D. R. Kaminski J. Barnitz R. A. Kurachi M. Gerdemann U. Yates K. B. (2016). The Epigenetic Landscape of T Cell Exhaustion. Science 354 (6316), 1165–1169. 10.1126/science.aae0491 27789799 PMC5497589

[B48] Spizzo R. Almeida M. I. Colombatti A. Calin G. A. (2012). Long Non-coding RNAs and Cancer: a New Frontier of Translational Research? Oncogene 31 (43), 4577–4587. 10.1038/onc.2011.621 22266873 PMC3433647

[B49] Sung H. Ferlay J. Siegel R. L. Laversanne M. Soerjomataram I. Jemal A. (2021). Global Cancer Statistics 2020: GLOBOCAN Estimates of Incidence and Mortality Worldwide for 36 Cancers in 185 Countries. CA A Cancer J. Clin. 71 (3), 209–249. 10.3322/caac.21660 33538338

[B50] Tamang S. Acharya V. Roy D. Sharma R. Aryaa A. Sharma U. (2019). SNHG12: An LncRNA as a Potential Therapeutic Target and Biomarker for Human Cancer. Front. Oncol. 9, 901. 10.3389/fonc.2019.00901 31620362 PMC6759952

[B51] Trowsdale J. Knight J. C. (2013). Major Histocompatibility Complex Genomics and Human Disease. Annu. Rev. Genom. Hum. Genet. 14, 301–323. 10.1146/annurev-genom-091212-153455 PMC4426292 23875801

[B52] Tsai M.-C. Manor O. Wan Y. Mosammaparast N. Wang J. K. Lan F. (2010). Long Noncoding RNA as Modular Scaffold of Histone Modification Complexes. Science 329 (5992), 689–693. 10.1126/science.1192002 20616235 PMC2967777

[B53] Vishnubalaji R. Alajez N. M. (2021). Epigenetic Regulation of Triple Negative Breast Cancer (TNBC) by TGF-β Signaling. Sci. Rep. 11 (1), 15410. 10.1038/s41598-021-94514-9 34326372 PMC8322425

[B54] Wang H. Huo X. Yang X.-R. He J. Cheng L. Wang N. (2017). STAT3-mediated Upregulation of lncRNA HOXD-AS1 as a ceRNA Facilitates Liver Cancer Metastasis by Regulating SOX4. Mol. Cancer 16 (1), 136. 10.1186/s12943-017-0680-1 28810927 PMC5558651

[B55] Wang Z. Yang B. Zhang M. Guo W. Wu Z. Wang Y. (2018). lncRNA Epigenetic Landscape Analysis Identifies EPIC1 as an Oncogenic lncRNA that Interacts with MYC and Promotes Cell-Cycle Progression in Cancer. Cancer Cell 33 (4), 706–720.e9. 10.1016/j.ccell.2018.03.006 29622465 PMC6143179

[B56] Wang Z.-l. Wang Z. Li G.-z. Wang Q.-w. Bao Z.-s. Zhang C.-b. (2019). Immune Cytolytic Activity Is Associated with Genetic and Clinical Properties of Glioma. Front. Immunol. 10, 1756. 10.3389/fimmu.2019.01756 31428092 PMC6688525

[B57] Wang M. Zheng S. Li X. Ding Y. Zhang M. Lin L. (2020). Integrated Analysis of lncRNA-miRNA-mRNA ceRNA Network Identified lncRNA EPB41L4A-AS1 as a Potential Biomarker in Non-small Cell Lung Cancer. Front. Genet. 11, 511676. 10.3389/fgene.2020.511676 33193600 PMC7530329

[B58] Wei Y. Dong S. Zhu Y. Zhao Y. Wu C. Zhu Y. (2019). DNA Co-methylation Analysis of lincRNAs across Nine Cancer Types Reveals Novel Potential Epigenetic Biomarkers in Cancer. Epigenomics 11 (10), 1177–1190. 10.2217/epi-2018-0138 31347388

[B59] Weigelin B. Krause M. Friedl P. (2011). Cytotoxic T Lymphocyte Migration and Effector Function in the Tumor Microenvironment. Immunol. Lett. 138 (1), 19–21. 10.1016/j.imlet.2011.02.016 21333682

[B60] Wilkerson M. D. Hayes D. N. (2010). ConsensusClusterPlus: a Class Discovery Tool with Confidence Assessments and Item Tracking. Bioinformatics 26 (12), 1572–1573. 10.1093/bioinformatics/btq170 20427518 PMC2881355

[B61] Xu M. Chen X. Lin K. Zeng K. Liu X. Xu X. (2019). lncRNA SNHG6 Regulates EZH2 Expression by Sponging miR-26a/b and miR-214 in Colorectal Cancer. J. Hematol. Oncol. 12 (1), 3. 10.1186/s13045-018-0690-5 30626446 PMC6327409

[B62] Xu D. Wang L. Pang S. Cao M. Wang W. Yu X. (2021). The Functional Characterization of Epigenetically Related lncRNAs Involved in Dysregulated CeRNA-CeRNA Networks across Eight Cancer Types. Front. Cell Dev. Biol. 9, 649755. 10.3389/fcell.2021.649755 34222227 PMC8247484

[B77] Xu J. Wang Z. Li S. Chen J. Zhang J. Jiang C. (2018). Combinatorial Epigenetic Regulation of Non-coding RNAs has Profound Effects on Oncogenic Pathways in Breast Cancer Subtypes. Brief Bioinform. 19 (1), 52–64. 10.1093/bib/bbw099 27742663

[B63] Yang Z. Xu F. Wang H. Teschendorff A. E. Xie F. He Y. (2021). Pan-cancer Characterization of Long Non-coding RNA and DNA Methylation Mediated Transcriptional Dysregulation. EBioMedicine 68, 103399. 10.1016/j.ebiom.2021.103399 34044218 PMC8245911

[B64] Ying K. Wang L. Long G. Lian C. Chen Z. Lin W. (2020). ACTA2‐AS1 Suppresses Lung Adenocarcinoma Progression via Sequestering miR‐378a‐3p and miR‐4428 to Elevate SOX7 Expression. Cell Biol. Int. 44 (12), 2438–2449. 10.1002/cbin.11451 32808728

[B65] Li Y. Jiang T. Zhou W. Li J. Li X. Wang Q. (2020). Pan-Cancer Characterization of Immune-Related lncRNAs Identifies Potential Oncogenic Biomarkers. Nat. Commun. 11 (1), 1000. 10.1038/s41467-020-14802-2 32081859 PMC7035327

[B66] Zhang G. Li S. Lu J. Ge Y. Wang Q. Ma G. (2018). LncRNA MT1JP Functions as a ceRNA in Regulating FBXW7 through Competitively Binding to miR-92a-3p in Gastric Cancer. Mol. Cancer 17 (1), 87. 10.1186/s12943-018-0829-6 29720189 PMC5930724

[B67] Zhang C. Ren X. Zhang W. He L. Qi L. Chen R. (2020). Prognostic and Clinical Significance of Long Non-coding RNA SNHG12 Expression in Various Cancers. Bioengineered 11 (1), 1112–1123. 10.1080/21655979.2020.1831361 33124951 PMC8291808

[B75] Zhang H. Bian C. Tu S. Yin F. Guo P. Zhang J. (2021a). Integrated Analysis of lncRNA-miRNA-mRNA ceRNA Network in Human Aortic Dissection. BMC Genomics 22 (1), 724. 10.1186/s12864-021-08012-3 34620091 PMC8495997

[B68] Zhang Z. Yan C. Li K. Bao S. Li L. Chen L. (2021b). Pan-cancer Characterization of lncRNA Modifiers of Immune Microenvironment Reveals Clinically Distinct De Novo Tumor Subtypes. npj Genom. Med. 6 (1), 52. 10.1038/s41525-021-00215-7 34140519 PMC8211863

[B69] Zhao H. Zhang X. Frazão J. B. Condino-Neto A. Newburger P. E. (2013). HOX Antisense lincRNA HOXA-AS2 Is an Apoptosis Repressor in allTransretinoic Acid Treated NB4 Promyelocytic Leukemia Cells. J. Cell. Biochem. 114 (10), 2375–2383. 10.1002/jcb.24586 23649634 PMC3999165

[B70] Zheng R. Zheng M. Wang M. Lu F. Hu M. (2021). Identification of a Prognostic Long Noncoding RNA Signature in Lung Squamous Cell Carcinoma: a Population-Based Study with a Mean Follow-Up of 3.5 Years. Arch. Public Health 79 (1), 61. 10.1186/s13690-021-00588-2 33910626 PMC8082628

[B71] Zhou R. J. Lv H. Z. (2019). Knockdown of ACTA2AS1 Promotes Liver Cancer Cell Proliferation, Migration and Invasion. Mol. Med. Rep. 19 (3), 2263–2270. 10.3892/mmr.2019.9856 30664183

