# Supplementary material for: Pan-Cancer Methylated Dysregulation of Long Non-coding RNAs Reveals Epigenetic Biomarkers
Source: Front Cell Dev Biol. 2022 May 27;10:882698. doi: 10.3389/fcell.2022.882698 (PMC9200062; doi:10.3389/fcell.2022.882698)
Supplement: Supplementary file 7 [file DataSheet1.docx]

Supplementary Material

#
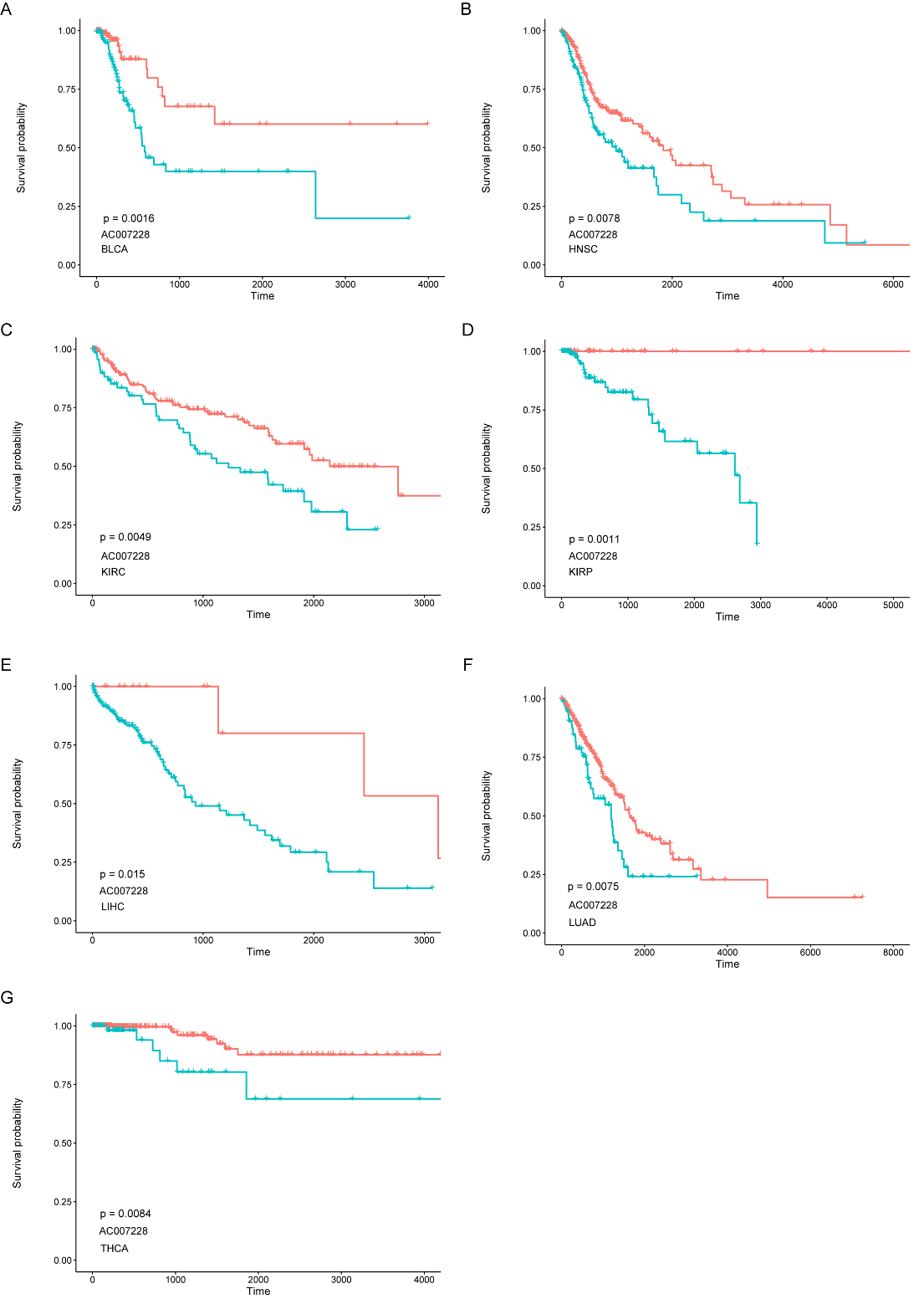


**Supplementary Figure S1.** The Kaplan-meier curves of AC007228 in each cancer. The red line represents the high expression group, the blue line represents the low expression group. And the "+" on the lines represents the patients who are lost to follow-up. At this time, the number of patients decreases but the overall survival rate does not decrease.

**
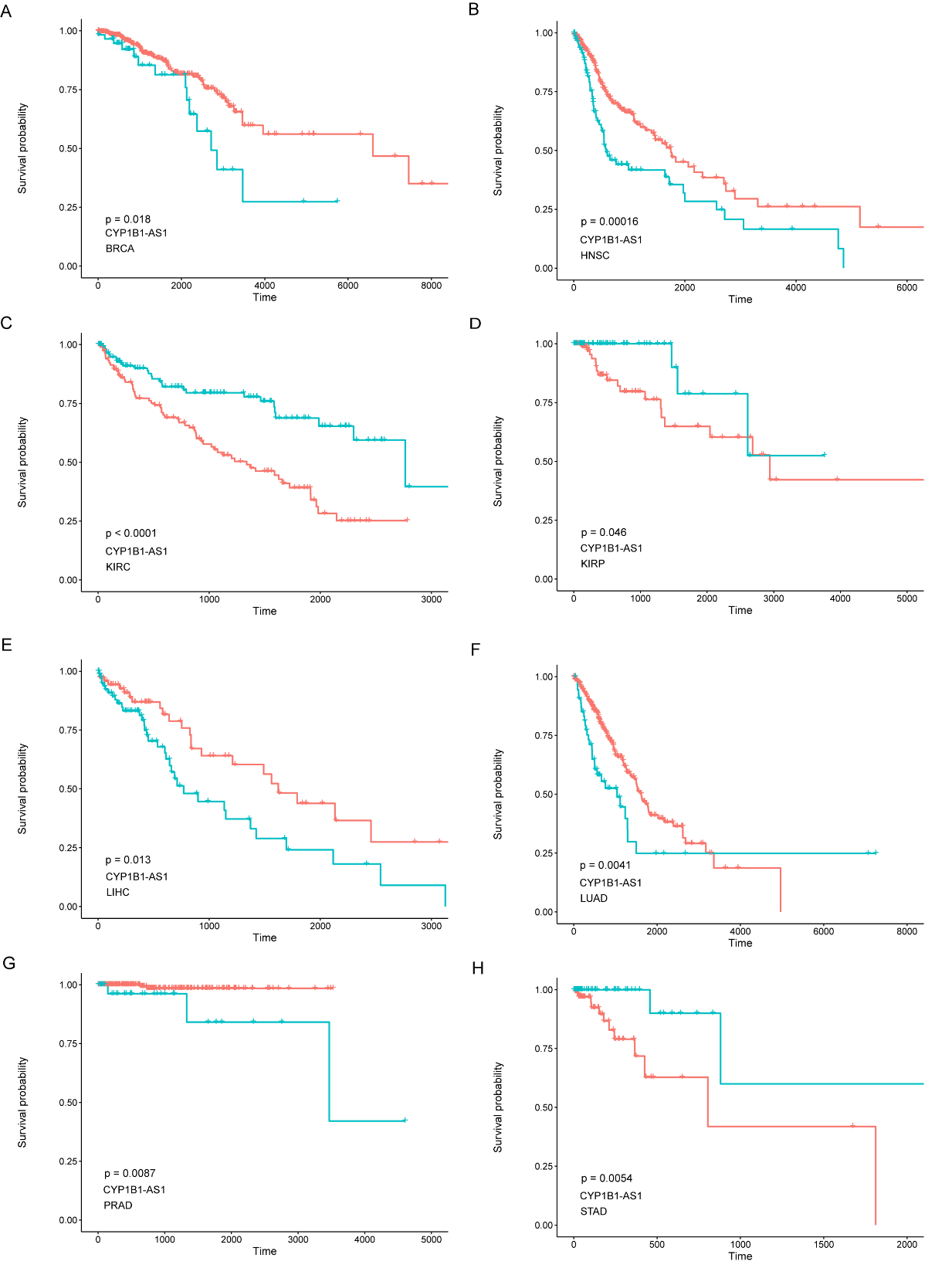
**

**Supplementary Figure S2.** The Kaplan-meier curves of CYP1B1-AS1 in each cancer. The red line represents the high expression group, the blue line represents the low expression group. And the "+" on the lines represents the patients who are lost to follow-up. At this time, the number of patients decreases but the overall survival rate does not decrease.

**
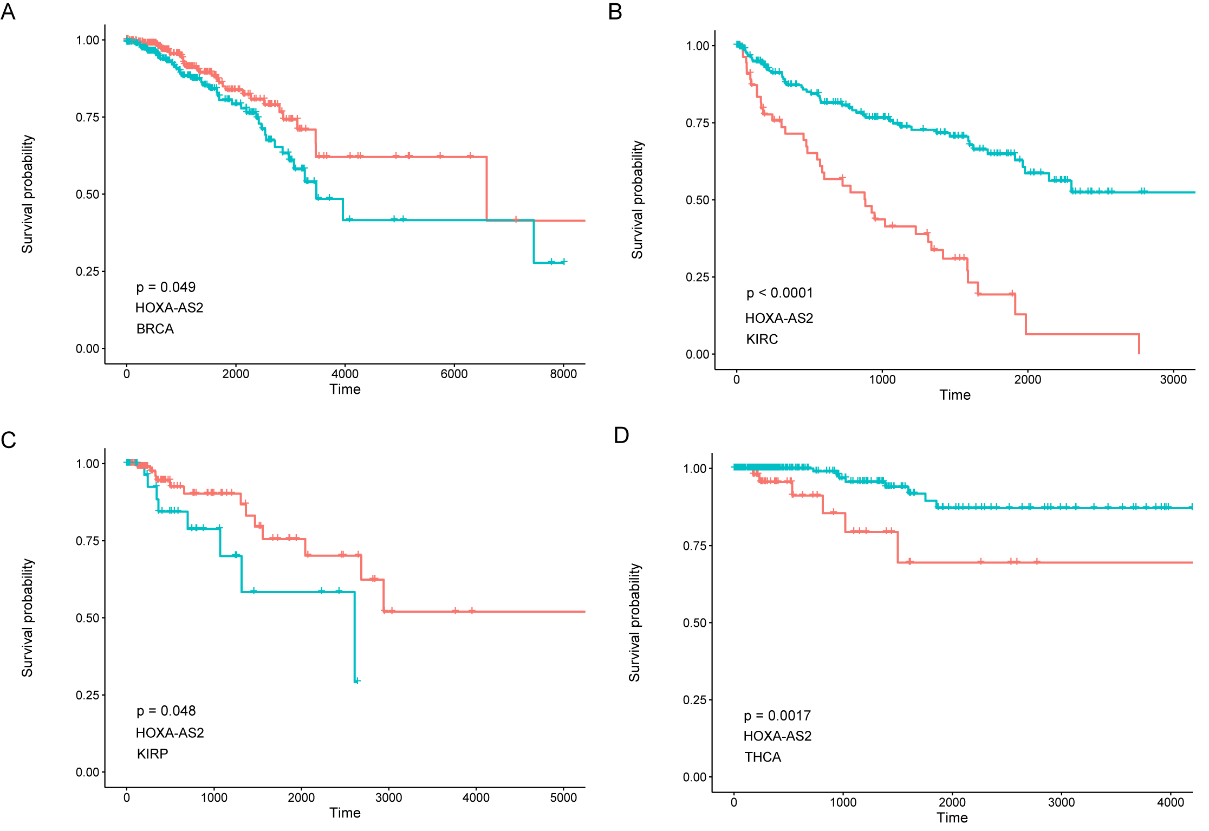
**

**Supplementary Figure S3.** The Kaplan-meier curves of HOXA-AS2 in each cancer. The red line represents the high expression group, the blue line represents the low expression group. And the "+" on the lines represents the patients who are lost to follow-up. At this time, the number of patients decreases but the overall survival rate does not decrease.

**
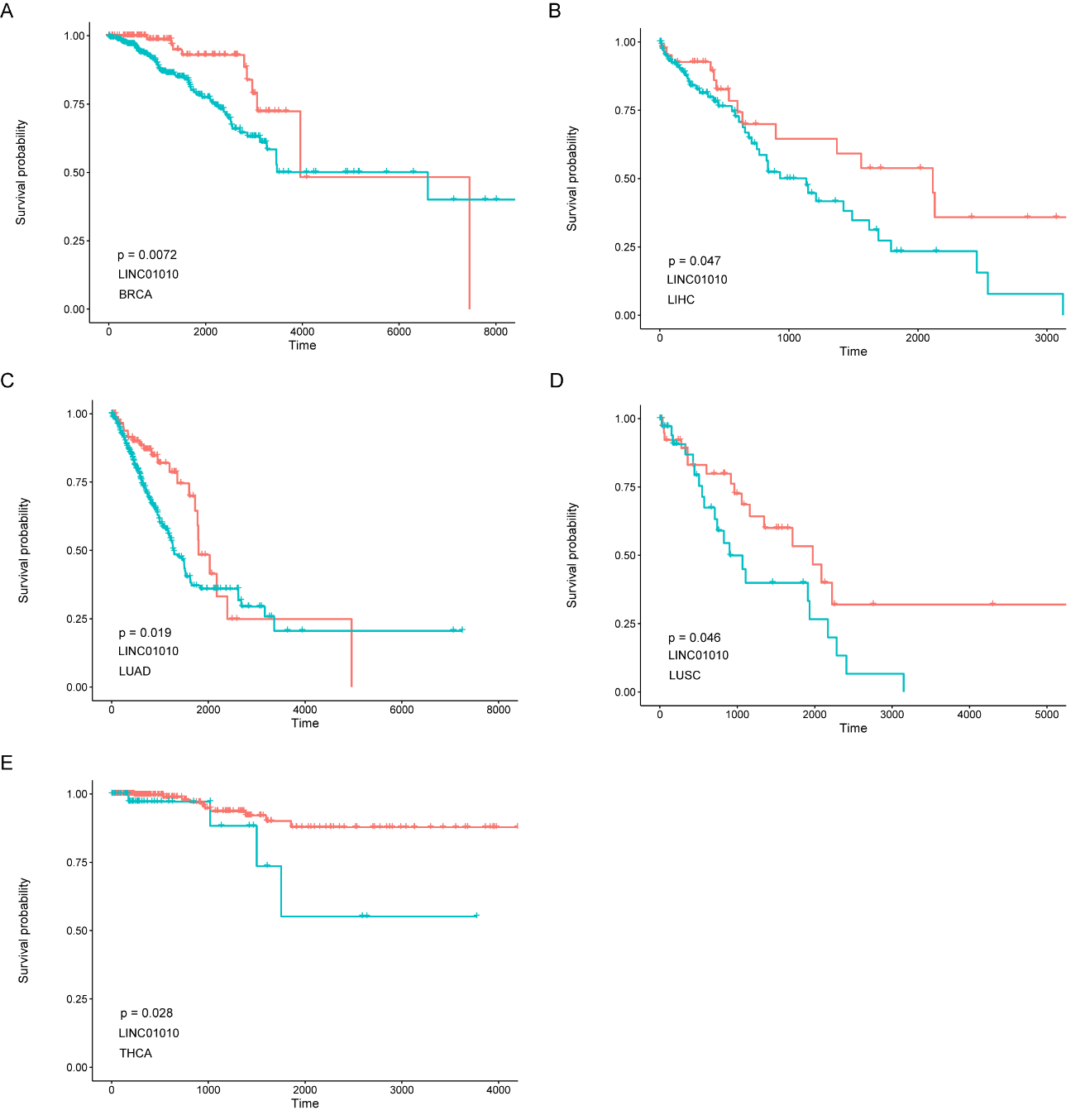
**

**Supplementary Figure S4.** The Kaplan-meier curves of LINC01010 in each cancer. The red line represents the high expression group, the blue line represents the low expression group. And the "+" on the lines represents the patients who are lost to follow-up. At this time, the number of patients decreases but the overall survival rate does not decrease.

**Supplementary Table Legends**

**Supplementary Table S1.** The number of samples and lncRNAs for each cancer in expression data.

**Supplementary Table S2.** The number of samples and mRNAs for each cancer in expression data.

**Supplementary Table S3.** The number of ceRNAs for each cancer.

**Supplementary Table S4.** The specific DMlncs of each cancer.

**Supplementary Table S5.** The functions of specific DMlncs of each cancer.

**Supplementary Table S6.** The number of differentially expressed lncRNAs for each cancer.
